# TNFSF14/LIGHT promotes cardiac fibrosis and atrial fibrillation vulnerability via PI3Kγ/SGK1 pathway-dependent M2 macrophage polarisation

**DOI:** 10.1186/s12967-023-04381-3

**Published:** 2023-08-14

**Authors:** Yirong Wu, Siyao Zhan, Lian Chen, Mingrui Sun, Miaofu Li, Xuanting Mou, Zhen Zhang, Linhao Xu, Yizhou Xu

**Affiliations:** 1 Department of Cardiology, Affiliated Hangzhou First People’s Hospital, Zhejiang University School of Medicine, Zhejiang, 310006 China; 2 Translational Medicine Research Center, Affiliated Hangzhou First People’s Hospital, Zhejiang University School of Medicine, Zhejiang, 310006 China; 3 Department of Orthopedics, Affiliated Hangzhou First People’s Hospital, Zhejiang University School of Medicine, Zhejiang, 310006 China

**Keywords:** Atrial fibrillation, Cardiac fibrosis, Tumour necrosis factor superfamily protein 14

## Abstract

**Background:**

Tumour necrosis factor superfamily protein 14 (TNFSF14), also called LIGHT, is an important regulator of immunological and fibrosis diseases. However, its specific involvement in cardiac fibrosis and atrial fibrillation (AF) has not been fully elucidated. The objective of this study is to examine the influence of LIGHT on the development of myocardial fibrosis and AF.

**Methods:**

PCR arrays of peripheral blood mononuclear cells (PBMCs) from patients with AF and sinus rhythm was used to identify the dominant differentially expressed genes, followed by ELISA to evaluate its serum protein levels. Morphological, functional, and electrophysiological changes in the heart were detected in vivo after the tail intravenous injection of recombinant LIGHT (rLIGHT) in mice for 4 weeks. rLIGHT was used to stimulate bone marrow-derived macrophages (BMDMs) to prepare a macrophage-conditioned medium (MCM) in vitro. Then, the MCM was used to culture mouse cardiac fibroblasts (CFs). The expression of relevant proteins and genes was determined using qRT-PCR, western blotting, and immunostaining.

**Results:**

The mRNA levels of LIGHT and TNFRSF14 were higher in the PBMCs of patients with AF than in those of the healthy controls. Additionally, the serum protein levels of LIGHT were higher in patients with AF than those in the healthy controls and were correlated with left atrial reverse remodelling. Furthermore, we demonstrated that rLIGHT injection promoted macrophage infiltration and M2 polarisation in the heart, in addition to promoting atrial fibrosis and AF inducibility in vivo, as detected with MASSON staining and atrial burst pacing respectively. RNA sequencing of heart samples revealed that the PI3Kγ/SGK1 pathway may participate in these pathological processes. Therefore, we confirmed the hypothesis that rLIGHT promotes BMDM M2 polarisation and TGB-β1 secretion, and that this process can be inhibited by PI3Kγ and SGK1 inhibitors in vitro. Meanwhile, increased collagen synthesis and myofibroblast transition were observed in LIGHT-stimulated MCM-cultured CFs and were ameliorated in the groups treated with PI3Kγ and SGK1 inhibitors.

**Conclusion:**

LIGHT protein levels in peripheral blood can be used as a prognostic marker for AF and to evaluate its severity. LIGHT promotes cardiac fibrosis and AF inducibility by promoting macrophage M2 polarisation, wherein PI3Kγ and SGK1 activation is indispensable.

**Supplementary Information:**

The online version contains supplementary material available at 10.1186/s12967-023-04381-3.

## Background

Atrial fibrillation (AF) is a prevalent arrhythmia affecting approximately 2% of the global population and leading to high morbidity and mortality rates [1]. Recent studies have highlighted that during AF, the immune system changes dramatically interacting with both the microenvironment and the cells responsible for initiating and maintaining AF. Moreover, pathological changes associated with AF, including fibrosis, act both as outcomes and positive regulators of immune remodelling, which further reinforces AF maintenance [2, 3]. However, the precise mechanisms underlying AF remain elusive.

The tumour necrosis factor (TNF) superfamily of cytokines plays important roles in fundamental immunological processes and AF pathogenesis [4, 5]. However, most studies has only focused on TNF-α, masking the essential role of other TNF superfamily members in the occurrence of AF. TNFSF14, also called LIGHT or CD258, is a type II transmembrane protein expressed primarily on activated T lymphocytes and other immune cells [6]. LIGHT plays an important role in immune responses and can exist in a soluble form produced by proteolytic cleavage. Its biological effects primarily stem from its binding to two receptors within the TNF receptor superfamily, namely, the herpesvirus entry mediator (HVEM or TNFRSF14) and the lymphotoxin β receptor (LTβR) which are widely expressed in various cell types, including macrophages, dendritic cells, neutrophils, fibroblastic reticular cells, and T and B lymphocytes [7, 8].Moreover, LIGHT has been widely demonstrated as an important regulator of immunological and fibrotic diseases, including eosinophilic oesophagitis [9, 10], hepatic steatosis and fibrosis [11, 12], rheumatoid arthritis [13], and unilateral ureteral obstruction-induced renal fibrosis [14].

However, few studies have related LIGHT and its receptors to cardiovascular disease. All that is known so far is that LIGHT and its receptors have been significantly overexpressed in multiple experimental and clinical models of heart failure [15]. Several clinical studies have indicated that circulating LIGHT levels or HVEM expressed in the peripheral blood can represent the severity and predict therapeutic outcomes of several diseases, including hypertension, stable coronary artery disease, and heart failure [16–18]. These preliminary results suggest a potential role of LIGHT and its receptors in cardiovascular diseases; however, this has not yet been confirmed.

Using in vivo and in vitro experiments, this study aimed to investigate, using in vivo and in vitro experiments, the direct role of LIGHT in AF occurrence and myocardial fibrosis and determine whether macrophages participate in the LIGHT-induced pathological process.

## Methods

### Reagents and antibodies

The mouse recombinant LIGHT protein (rLIGHT, cat#HY-P73837), PI3Kγ specific inhibitor (Eganelisib, IPI549, cat# HY-100716), and SGK1 inhibitor (GSK650394, cat#HY-15192) were purchased from MedChemExpress (MCE, New Jersey, USA). The primary antibodies anti-phospho-Akt (Ser473) (cat#4060) and anti-Akt (cat#4691)used for western blotting were purchased from Cell Signalling Technology (Danvers, Essex County, MA, USA); anti-PIK3CG (cat#A0266), anti-SGK1 (cat#A3936), and anti-phospho-Smad2/Smad3 (cat#AP1343) were purchased from ABclonal (Wuhan, China); anti-Smad2 (cat#ET1604-2), anti-Smad3 (cat#ET1607-4) and anti-PI3K p85α (cat# ET1608-70)were purchased from HUABIO (Hangzhou, China); and anti-F4/80 (cat#28463-1-AP), anti-CD3 (cat#17617-1-AP), anti-CD163 (cat#16646-1-AP), anti-ARG1 (cat#66129-1-Ig), anti-CD206(cat#60143-1-Ig), anti-iNOS (cat#18985-1-AP), anti-MCP1 (cat#66272-1-Ig), anti-TGFβ1 (cat#21898-1-AP), anti-collagen I (cat#14695-1-AP), anti-collagen III (cat#68320-1-Ig), anti-α-SMA (cat#14395-1-AP), anti-β actin (cat#20536-1-AP), and anti-GAPDH(cat#10494-1-AP) were purchased from Proteintech (Wuhan, China).

### Data collection from public database, data processing, and bioinformatic analysis

Using the keyword ‘Atrial Fibrillation’, we downloaded two microarray expression profiles of human AF, including the GSE14975 and GSE79768 datasets, which altogether includes 19 atrial AF samples and 17 sinus rhythm atrial samples, from the Gene Expression Omnibus (GEO) database (https://www.ncbi.nlm. nih.gov/geo/). The platform of GSE14975 and GSE79768 is GPL570 [HG-U133_Plus_2] Affymetrix Human Genome U133 Plus 2.0 Array. The ‘hugene10sttranscriptcluster.db’ R package was used for geneID transformation. After merging all microarray data, the batch effects were adjusted through the application of the ‘combat’ function available in the ‘SVA’ package in R. We then screened the genes differentially expressed between AF and sinus rhythm (SR) patients in the merged dataset using the ‘limma’ R package. Genes were defined as differentially expressed between the AF and SR samples when the P-value was < 0.05 and fold change was > 1.5 or < 0.67. GO and KEGG pathway enrichment analyses of differentially expressed genes (DEGs) in this combined dataset were performed using the online Sangerbox platform [19].

### Immune infiltration analysis

To quantify the proportions of immune cells in AF and SR atrial samples, we used the Xcell algorithm, which is a novel gene signature-based strategy used to identify various immune and stromal cell types [20]. Differences in cell types between the AF group and SR control were evaluated using the t-test, and cutoff values for significance were set at p < 0.05. The ESTIMATE method [21] was used to infer the fractions of stromal and immune cells in atrial tissue samples. The ESTIMATE score was determined by summing the immune and stromal cell scores, which respectively reflect the relative abundance of immune and stromal components within the tissue.

### Analysis of single cell sequencing data

We used single-nucleus RNA sequencing data of approximately 592,689 nuclei in the left ventricle samples from 11 dilated cardiomyopathy, 15 hypertrophic cardiomyopathy, and 16 non-failing hearts from single-nucleus profiling of human dilated and hypertrophic cardiomyopathy. Additionally, we used the single-nucleus RNA sequencing data of 31,021 nuclei from two peripheral blood mononuclear cell (PBMC) samples. Both single-nucleus RNA sequencing data were derived from the single-cell portal database (https://singlecell.broadinstitute.org).

### Clinical sample collection and processing and PBMC separation

Peripheral blood samples were obtained from 16 volunteers with SR [three for both PCR array analysis and enzyme-linked immunosorbent assay (ELISA) analysis and 13 for ELISA analysis only] and 30 patients with AF (four for PCR array analysis and 26 patients for ELISA analysis) in the cardiology department at the Affiliated Hangzhou First People's Hospital, Zhejiang University School of Medicine (Hangzhou, China). Patients with left ventricle ejection fraction (LVEF) reduced heart failure, chronic respiratory diseases, chronic kidney disease, alcohol abuse, malignant tumours, or a history of drug use were excluded from the study. Informed consent was obtained from all subjects, and the experimental procedures were approved by the Research Ethics Committee of the Affiliated Hangzhou First People's Hospital, Zhejiang University School of Medicine. The approval date was July 21st, 2022. Peripheral blood was stored in ethylenediaminetetraacetic acid coated tubes. All samples were centrifuged at 3,000 rpm and 4 °C for 10 min within 12 h of collection. Peripheral blood plasma was collected for use in the LIGHT enzyme-linked immunosorbent assay (ELISA). Mononuclear cells were isolated from remnant peripheral blood via Ficoll separation as previously described [22]. Specifically, blood was gently layered on a Ficoll gradient and centrifuged for 30 min at 300 × g. The PBMCs were then collected and washed with phosphate buffered saline.

### Flow cytometry(FCM)

After PBMC separation and cell counting, cells for live-dead cell staining (10^6^ cells per tube) were primarily labelled with by the Zombie Green™ Fixable Viability Kit (BioLegend, CA, USA, cat#423111) for 15 min protected from light. After washing with staining buffer (BioLegend, CA, USA, cat#420201), membrane receptors staining was performed using Brilliant Violet 421™ anti-human CD14 (BioLegend, CA, USA, cat#325628), Brilliant Violet 605™ anti-human CD3 (BioLegend, CA, USA, cat#317322), PE anti-human LTβR (BioLegend, CA, USA, cat#322008), PE/CY7 anti-human HVEM (BioLegend, CA, USA, cat#318810) and APC anti-human LIGHT (BioLegend, CA, USA, cat#392708) at 4 °C for 20 min protected from light. After washing twice with staining buffer, PBMCs were resuspend with in 250 µL staining buffer.

The bone marrow-derived macrophages (BMDMs) for FCM analysis were digested with trypsin and then collected, washed twice with PBS, and suspended with 100 µL staining buffer (10^6^ cells per tube). BMDMs were first labelled with APC anti-mouse F4/80 (BioLegend, cat#123116) at 4 °C for 20 min protected from light. They were then labelled with Brilliant Violet 421™ anti-mouse CD206 (BioLegend, cat#141717) at 4 °C for 20 min protected from light followed by membrane breaking and fixing. After washing twice with staining buffer, the BMDMs were resuspend in 250 µL staining buffer.

Flow cytometry was performed using a Beckman CytoFLEX instrument (Beckman Coulter Inc., USA). All data evaluation was performed using FlowJo software.

### Animal experiments

C57BL/6 male mice, aged 6–8 weeks and weighing 20–22 g, were provided by Zhejiang Chinese Medical University. The animal experiments were conducted at the Experimental Animal Center of Zhejiang Chinese Medical University under registration number of IACUC-20220913-04. The approval date was September 13th, 2022. The mice were housed in a specific pathogen-free (SPF) facility, maintained at 22 °C with 12 h of light per day, and provided ad libitum access to food and water. After allowing the mice to acclimatize for 7 days, they were randomly assigned to one of two groups. The LIGHT group received a weekly dose of 10 µg rLIGHT via tail vein injection for 4 weeks, whereas the control group received a 0.9% normal saline solution based on a previously published study [12].

### Echocardiographic assessment of cardiac functions

Four weeks after rLIGHT injection, the mice were anaesthetised with isoflurane. Cardiac function of the left ventricle (LV) and left atrium (LA), including LVEF, LV fractional shortening (LVFS), LV internal dimensions at diastole/systole (LVIDd/LVIDs), and LA diameter, were evaluated using echocardiography with Vevo TM 2100 (Visual Sonics Inc., Ontario, Canada) in the transthoracic parasternal long-axis view.

### Intracardiac electrophysiological examination and induction of AF

Following echocardiographic assessment, the mice were anesthetized with pentobarbital sodium (30 mg/kg). Subcutaneous ECG electrodes were positioned in the limbs, and ECG data were recorded for 1 min. Subsequently, a 1/2 inch incision was made to the right of the midline, with the caudal terminus at the level of the clavicle. After separating the right jugular vein, the proximal end of the vein was tied with a 6–0 suture. A small incision was made along the longitudinal direction of the vein, then, atrial intracardiac electrograms were recorded by inserting a 1.1F octapolar catheter (iWorx Systems Inc., NH, USA) through the right jugular vein. The inducibility of AF was tested three times according to the protocol described by Verheule et al. [23]. The inducibility of atrial arrhythmias was tested by applying 2-s bursts using an automated stimulator that was integrated with the data acquisition software. The first 2-s burst had a cycle length (CL) of 40 ms, decreasing in each successive burst with a 2-ms decrement down to a CL of 20 ms. AF was defined as a period of rapid irregular atrial rhythm lasting at least 2 s. The electrode voltage was amplified using an iWorx IX-RA-834 multichannel recorder (iWorx Systems Inc., NH, USA) and recorded using the LabScribe2 software (iWorx).

### Histological staining

After the echocardiographic and electrophysiological assessments were both accomplished, the hearts were isolated and fixed in 4% paraformaldehyde for 24 h followed by gradual dehydration. The fixed heart tissues were embedded in paraffin and subjected to haematoxylin and eosin (H&E) and Masson’s trichrome. To label the macrophages and lymphocytes in the heart tissues of mice, antibodies against murine F4/80 and CD3 were used for immunohistochemical (IHC) staining. To label the tendency of monocytes infiltration and M1/2 macrophage polarisation, antibodies against murine MCP-1, CD163, CD206, and inducible nitric oxide synthase (iNOS) were used for IHC staining. Briefly, tissue sections were incubated with primary antibodies overnight at 4 °C, then incubated with secondary antibodies, followed by DAPI staining. All pathological staining and IHC sections were scanned using a digital slide scanner (Aperio Versa 8, Leica, Germany).

### RNA sequencing and bioinformatic analysis

A small portion of fresh myocardium (approximately 5–10 mg) from three hearts in each of the groups was used to extract total RNA. The mRNA-seq Library Prep kit (Vazyme, Nanjing, China, cat#NR612-02) was used to prepare a transcriptome library with at least 1 μg total RNA. Transcriptome sequencing and analysis were conducted by OE Biotech Co. Ltd. (Shanghai, China). Differential expression analysis was performed using the R package ‘DESeq2’. The thresholds for significant DEGs were set as p < 0.05 and log_2_ fold change (FC) values > 0.75. GO enrichment and KEGG pathway enrichment analyses of DEGs were performed using the Xiantao platform (https://www.xiantao.love/products).

### Cell isolation and culture

Under sterile conditions, bone marrow-derived macrophages (BMDMs) were isolated from 6–8-week-old mouse femur and tibia. The bones were flushed with Dulbecco’s modified Eagle’s medium (DMEM) supplemented with 10% FBS (Invitrogen, GIBCO). BMDMs were cultured in DMEM supplemented with 10% FBS (Invitrogen GIBCO), 100 U/mL penicillin–streptomycin (Invitrogen GIBCO), and 10 ng/L macrophage colony-stimulating hormone (M-CSF; Novoprotein, Suzhou, China, cat#C756) for 6 days.

Primary cardiac fibroblasts (CFs) were isolated from 3 day-old neonatal mice using collagenase type II (Worthington, Lakewood, NJ, USA, cat# LS004177) digestion [24]. Briefly, the hearts were excised, minced, and placed in spinner flasks containing 0.1% collagenase type II. The ventricles were repeatedly digested (10–15 min at 37 °C), and the cells released by the second to fifth digestions were pooled, pelleted, and resuspended in DMEM supplemented with 10% FBS (Invitrogen GIBCO) and 100 U/mL penicillin–streptomycin (Invitrogen GIBCO). The cells were plated for 45 min to allow CFs to preferentially attach, after which unattached cells were removed by aspiration and fresh medium was added. In this experiment, CFs were sub-cultured using trypsin and used within three passages.

### CCK8 assay

For drug cytotoxicity detection, BMDMs were seeded on 96-well plates followed by continuous stimulation with M-CSF for 6 days. Different concentrations of IPI549 (0 and 500 nM/L, and 1, 2, and 5 μM/L) or GSK650395 (0 μM/L, 5 μM/L, 10 μM/L, 20 μM/L and 40 μM/L) were used to pretreat BMDMs for 2 h, respectively. Then, the culture media were removed and replaced by DMEM supplemented with 10% FBS for 24 h. The CCK8 reagent (Dojindo, Kumamoto, Japan, Cat#CK04) was added according to the manufacturer’s protocol, and OD 450 was obtained from the plate reader.

### Preparation of macrophage-conditioned medium for CF treatment

After continuous stimulation with M-CSF for 6 days, rLIGHT (100, 200, or 300 ng/L) with M-CSF 10 ng/L was used to stimulate BMDMs simultaneously for 24 h. BMDMs were pre-treated with IPI549 (1 μM/L) or GSK650395 (20 μM/L) for 2 h, respectively, before treatment with rLIGHT. BMDM culture supernatants were collected after 24 h of BMDM stimulation to prepare a MCM. The MCM was then centrifuged at 2000 rpm at room temperature for 5 min, and the centrifugal supernatants were used to culture CFs at a 1:2 dilution ratio for 48 h.

### ELISA

Frozen human plasma samples were used to measure LIGHT levels. Mouse serum samples or BMDM supernatants were used to measure the levels of TGF-β1, IL-10, IL-4, TNF-α, and IL-1β. ELISA was performed using commercial kits [the Human LIGHT/LIGHT Quantikine ELISA Kit (R&D Systems Inc., Minneapolis, USA, cat#DLIT00), Mouse Transforming Growth Factor Beta 1 ELISA Kit (ABclonal, Wuhan, China, cat#RK0057), Mouse IL-10 ELISA Kit (ABclonal, cat#RK00016), Mouse IL-4 ELISA Kit (ABclonal, cat#RK00036), Mouse TNF-α ELISA Kit (Elabscience Biotechnology Co., Ltd, Wuhan, China, cat#E-EL-M3063), Mouse NT-proBNP ELISA Kit (Elabscience Biotechnology Co., Ltd, cat# E-EL-M0834c) and IL-1 beta Mouse ELISA Kit (Thermo Fisher Scientific Inc., Waltham, MA, USA, cat#88-7013-77)] according to the manufacturer’s instructions.

### Quantitative reverse transcription (qRT-PCR) and PCR array

Total cellular RNA and tissue RNA were isolated using the RNA-Quick Purification Kit and Tissue RNA Purification Kit Plus (Yishan, Shanghai, China, cat#ES-RN001), respectively, according to the manufacturer’s recommendations. The isolated RNA was quantified with the photometric method using a NanoDrop spectrophotometer (Thermo Fisher Scientific). Subsequently, 500 ng of total RNA was reverse-transcribed into cDNA using the HiFiScript cDNA synthesis kit (Cwbio, Taizhou, China, cat#CW2569M) following the manufacturer’s instructions. The predesigned primer PCR array for immune checkpoints and cytokines (wc-mR0276CP-H) was synthesized by Wcgene (Shanghai, China). The qRT-PCR primers utilized in this study were synthesized by Sangon Biotech (Shanghai, China), and the primer sequences can be found in Additional file 1: Table S1. For qRT-PCR, the UltraSYBR mixture (Cwbio, cat#CW2601H) and ABI 7500 real-time PCR detection system (Thermo Fisher Scientific) were used to obtain the comparative threshold cycle (Ct) value of the target genes. GAPDH was used as the internal control. The relative fold-change in gene expression was calculated using the 2^−ΔΔCt^ method.

### Western blot analysis and cell immunofluorescence

Proteins were obtained from BMDMs, CFs, and myocardial tissues. Cells or tissues were homogenized in RIPA buffer containing a protease and phosphatase inhibitor. After measuring the protein concentration, the proteins were separated by SDS-PAGE and transferred onto a polyvinylidene difluoride (PVDF) membrane (Millipore, Billerica, MA, USA). The membrane was then blocked with non-fat dry milk (5%) and incubated with primary antibodies, followed by secondary antibodies. Finally, the membrane was visualized using an ECL substrate (Thermo Fisher Scientific). After phospho-Akt or phospho-Smad2/3 detection, the membranes were stripped with stripping buffer (Beyotime, Shanghai, China, cat#P0025) and reblotted with antibodies against the anti-Akt, anti-Smad2 and Smad3, or β actin antibodies.

To conduct cell immunofluorescence, the cell monolayers were fixed with 4% paraformaldehyde followed by treatment with 0.3% Triton X-100. After three washes with phosphate-buffered saline, the cells were blocked with 5% bovine serum albumin for 40 min. The cells were then incubated with primary antibodies, followed by fluorescently labelled secondary antibodies and Hoechst. The final results were observed using a fluorescence microscope (Leica).

### Statistical analysis

The data from separate experiments were analysed using GraphPad Prism 8 Project software. Two-tailed unpaired t-test were employed to compare the means of two groups, while one-way ANOVA tests were used to compare the means of more than two groups. P-value < 0.05 was considered statistically significant. The results were graphically presented using GraphPad Prism 8.

## Results

### Inflammatory and immune responses are associated with AF occurrence and development

**Fig. 1:**
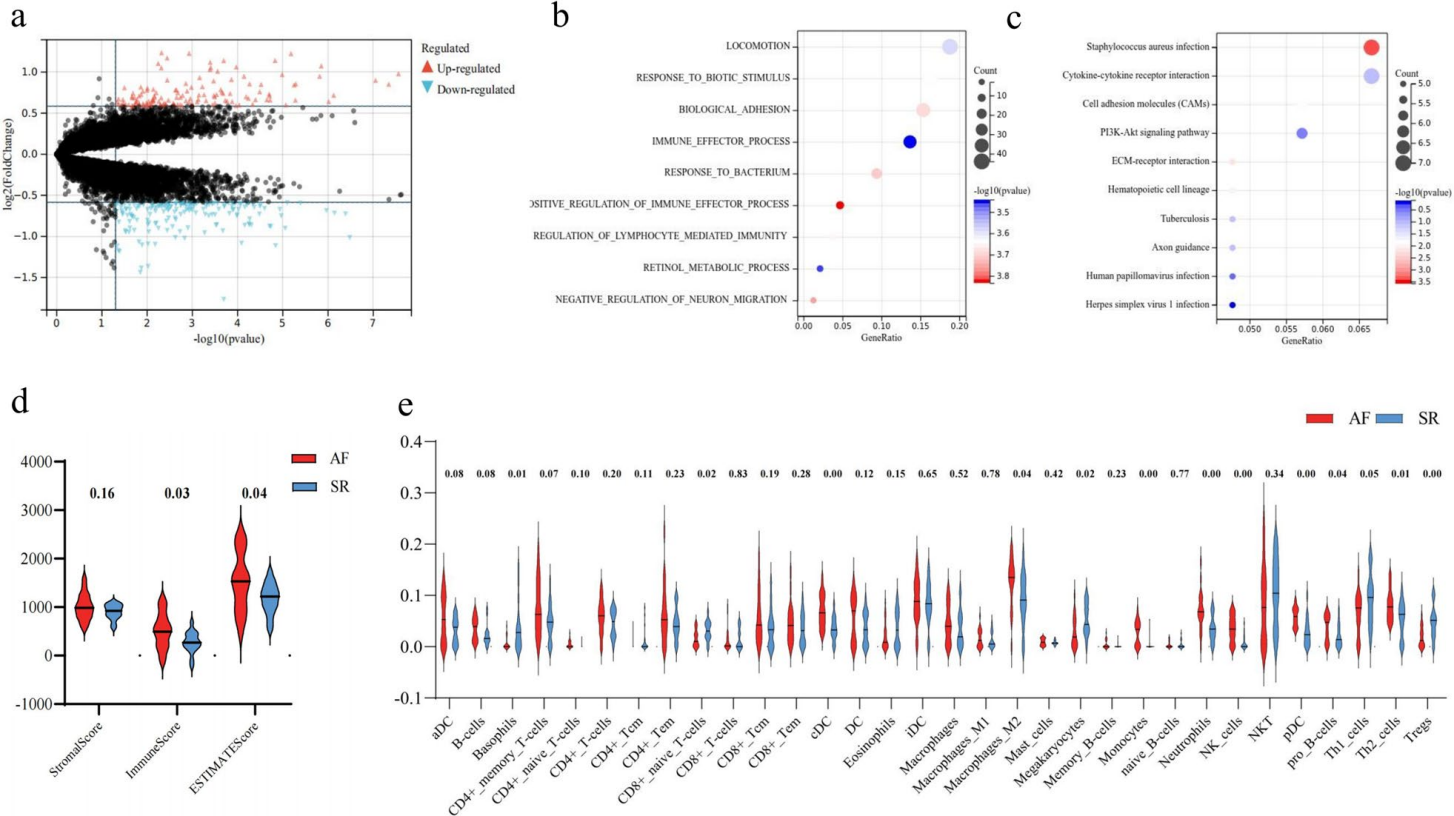
Bioinformatic and immune infiltration analyses of the merged dataset. **a** Volcano plot of 310 differentially expressed genes. **b** GO and **c** KEGG enrichment analyses of 310 differentially expressed genes. **d** ESTIMATE analysis of the merged dataset. **e** Xcell analysis of the merged dataset

Gene expression levels of the merged datasets were adjusted for batch effects and standardised. A comparison between the pre- and post-expression matrixes is presented in Supplementary Fig. 1a, b. After converting probes to gene symbols, 310 DEGs (136 upregulated and 174 downregulated) were confirmed. Volcano plots of the 310 DEGs were used in subsequent analyses (Fig. 1a). The following analysis demonstrated that immune related pathways like ‘immune effector process’ and ‘cytokine and cytokine receptor interaction’ were prominently enriched in the GO and KEGG enrichment analyses (Fig. 1b, c). Further immune infiltration analysis highlighted the inflammatory and immune responses in the AF group. Specifically, ESTIMATE analysis showed that both the immune and ESTIMATE scores were higher in AF group, and Xcell analysis further demonstrated that basophils, CD8^+^ naïve T cells, Th1/2, Treg, dendritic cells, M2 macrophages, mast cells, monocytes, neutrophils, megakaryocytes, natural killer (NK) cells, and NK-T cells tended to infiltrate the atrial tissues of patients with AF compared to those of individuals with SR. Thus, we could reasonably surmise that inflammatory and immune responses are associated with AF occurrence and development.

### LIGHT expression increases in patients with AF and is related to LA adverse remodelling

**Fig. 2:**
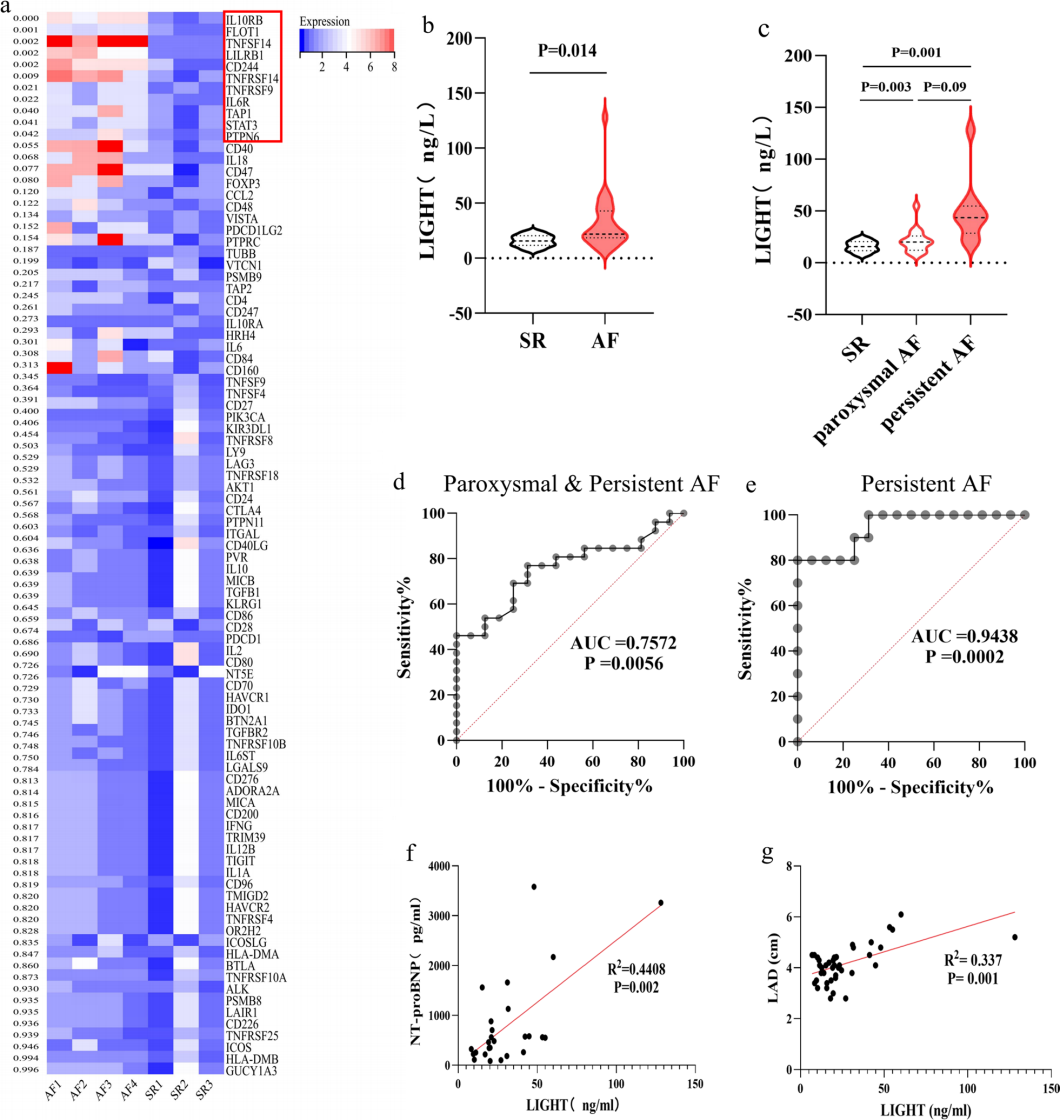
LIGHT expression is increased in patients with AF and related to LA adverse remodelling. **a** Heatmap of gene expression in the PBMCs of patients with AF and individuals with SR. **b** The plasma protein levels of LIGHT in patients with AF and SR. **c** Plasma protein levels of LIGHT in persistent and paroxysmal patients with AF and individuals with SR. **d** ROC curve of LIGHT in diagnosing all types of AF and **e** persistent AF. **f** The correlation between LIGHT protein levels and NT-proBNP. **g** The correlation between LIGHT protein levels and LAD. ROC: receiver operator characteristic; LAD: LA diameter

Since AF occurrence and development are associated with the inflammatory and immune responses, we conducted an ‘immune checkpoint and cytokine’ PCR array in the PBMCs of four patients with AF and three SR controls, who all without heart failure, severe organ dysfunction, or malignant tumours. Detailed baseline information is presented in Additional file 1: Table S2. Surprisingly, we discovered that multiple cytokines or receptors were significantly differentially expressed between the AF and SR groups (Fig. 2a). Among the differentially expressed genes, *LIGHT* and *TNFRSF14*, which are reciprocal ligands and receptors [7], were significantly overexpressed in the AF group, indicating that LIGHT could be a novel but crucial target in AF occurrence and development.

Next, expanded samples (n = 42) of patients with AF (n = 26) and corresponding SR controls (n = 16) were collected to investigate the relationship between LIGHT and AF (detailed baseline information is listed in Additional file 1: Table S3). The expression of secreted LIGHT detected using ELISA demonstrated that the levels of secreted LIGHT were significantly elevated in the AF group (Fig. 2b). Moreover, a more obvious increase was observed in the persistent AF group than in its paroxysmal counterpart when they were compared to the SR controls (Fig. 2c). The diagnostic values of secreted LIGHT in all types of AF and in persistent AF alone were evaluated using ROC curves (AUC = 0.7572 and 0.9428, respectively) (Fig. 2d, e). Further analysis of the relationship between secreted LIGHT levels and clinical characteristics revealed that LIGHT levels were closely related to LA adverse remodelling, manifested as a high correlation coefficient between LIGHT levels and NT-proBNP and LA diameter (Fig. 2f, g).

To exclude the confounding variables, univariate and multivariate logistic regression analyses were performed between patients with AF and SR controls. Ultivariate logistic regression analysis demonstrated that the secreted LIGHT level increased with the AF incidence with an OR = 1.129 (P = 0.018, 95% CI [1.021, 1.248]) (Additional file 1: Table S4). However, multivariate logistic regression analysis showed a nonsignificant influence on AF incidence (Additional file 1: Table S5), which may have been caused by relatively inadequate sample. Nevertheless, these findings are sufficient to suggest that secreted LIGHT is intricately connected with AF occurrence and that significantly elevated serum level of LIGHT may be a unique indicator in demonstrating LA structural remodelling.

### Cell population distribution of LIGHT and its receptors reveals the potential immunoregulatory activity

FCM analysis of LIGHT and its receptors in PBMCs revealed their expression heterogeneity among different cell populations. Similarly to those in previous studies [7, 15], TNFRSF14 and LTβR were mainly expressed in CD14^+^ cells in human peripheral blood (Additional file 1: Fig. S2a). However, LIGHT (membrane ligand) was also mainly expressed in CD14^+^ cells (Additional file 1: Fig. S2a), which is different from the results in peripheral blood analysed with scRNA sequence data. According to the scRNA sequence data, LIGHT tends to be expressed by lymphocytes or NK cells;LTβR are widely expressed in macrophages, dendritic cells, and other antigen-presenting cells while TNFRSF14 are widely expressed in all types of immune cells with siginificantly higher expression in CD16^+^ monocytes (Additional file 1: Fig. S2b, c).

With respect to the expression heterogeneity among different cell populations of LIGHT and its receptors within the myocardium, we conducted scRNA sequence data analysis of the dysfunctional myocardium. We found that LIGHT was mainly expressed in lymphocytes, whereas TNFRSF14 and LTβR were widely expressed in all types cells with relatively higher expression levels in macrophages (Additional file 1: Fig. S3a, b). This special expression heterogeneity between cell populations may indicate that LIGHT and its receptors are highly associated with immune cell activity. Furthermore, under myocardial injury, macrophages may become the dominant receptor cells during LIGHT stimulation, whereas lymphocytes may become the main secretion cells.

### LIGHT contributes to cardiac dysfunction and AF

**Fig. 3:**
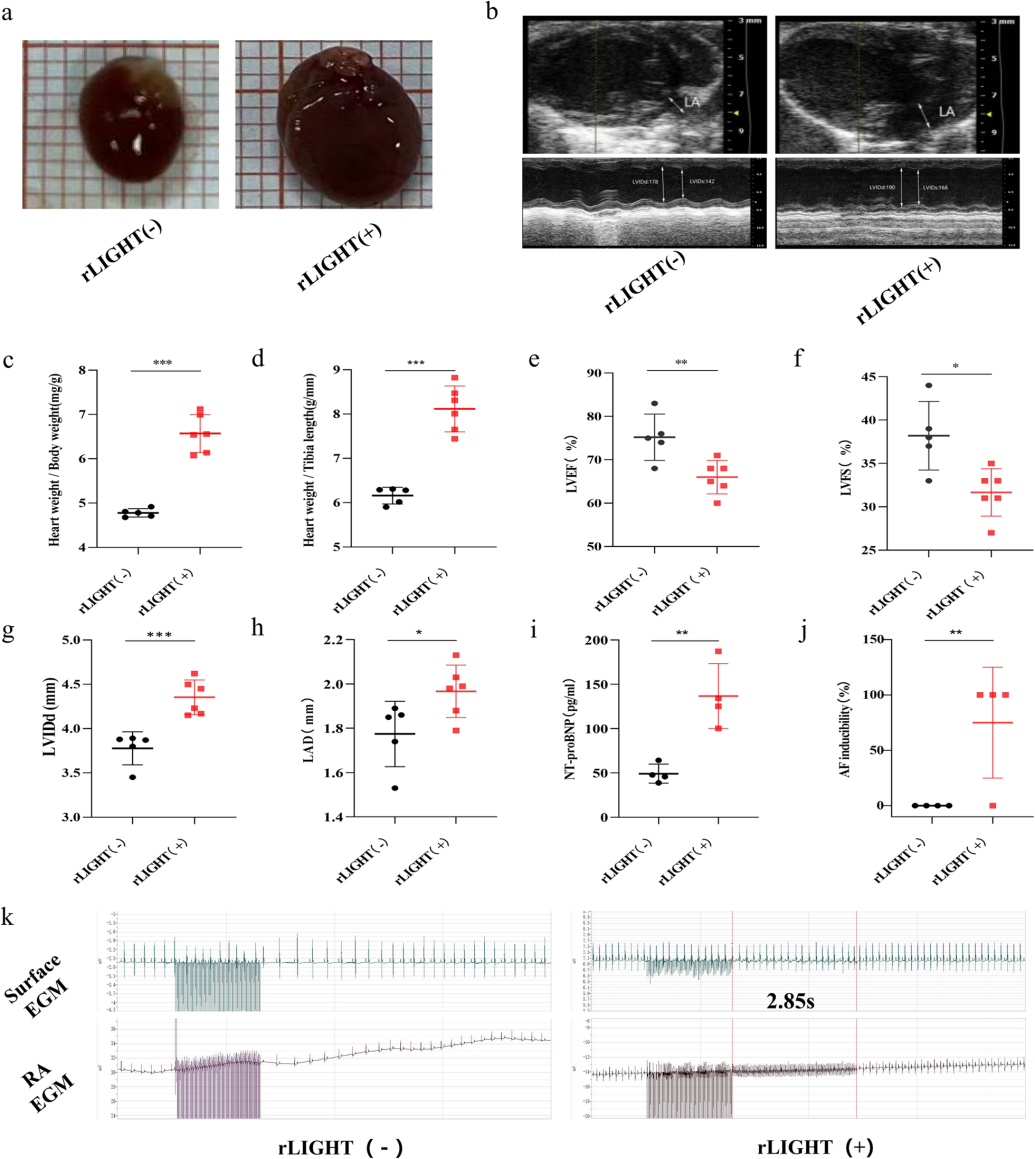
LIGHT contributes to cardiac dysfunction and AF. **a** Representative images of cardiac morphology with or without LIGHT treatment. **b** Typical echocardiography of the heart with or without LIGHT treatment. **c**–**j** Ratio of heart weight/body weight, heart weight/tibia length, NT-proBNP, LVEF, LVFS, LVIDd, and LAD and AF inducibility in the control and LIGHT (+) groups. (k) Representative images of surface and RA EGM in the LIGHT (−) and LIGHT (+) groups. *p < 0.05, **p < 0.01. RA: Right atria; EGM: Electrogram, NT-proBNP: N-terminal pro brain natriuretic peptide

To explore the function of LIGHT in vivo, we used mouse recombinant LIGHT protein to simulate high circulating LIGHT levels. LIGHT-treated mice showed enlarged hearts compared to those of the control mice (Fig. 3a). In addition, the ratios of heart weight/body weight and heart weight/tibia length were higher than those in the control mice (Fig. 3c, d). Echocardiography revealed that LVEF and LV fractional shortening (FS) decreased in the LIGHT group (Fig. 3b, e, and f), whereas LVIDd and LAD increased in the LIGHT group (Fig. 3g, h), indicating that both atrial and ventricular functions were impaired in the LIGHT group. Moreover, NT-proBNP, an important indicator of HF, was significantly upregulated compared to that in the control group (Fig. 3i). Moreover, to test AF inducibility in the LIGHT group, we utilised the intracardiac electrophysiology stimulating system and found that three of four mice had undergone AF occurrence during right atrial burst pacing (Fig. 3j, k). These investigations demonstrated the potential detrimental effects of LIGHT in promoting cardiac dysfunction and AF.

### LIGHT contributes to monocyte migration and macrophage M2 polarization

**Fig. 4:**
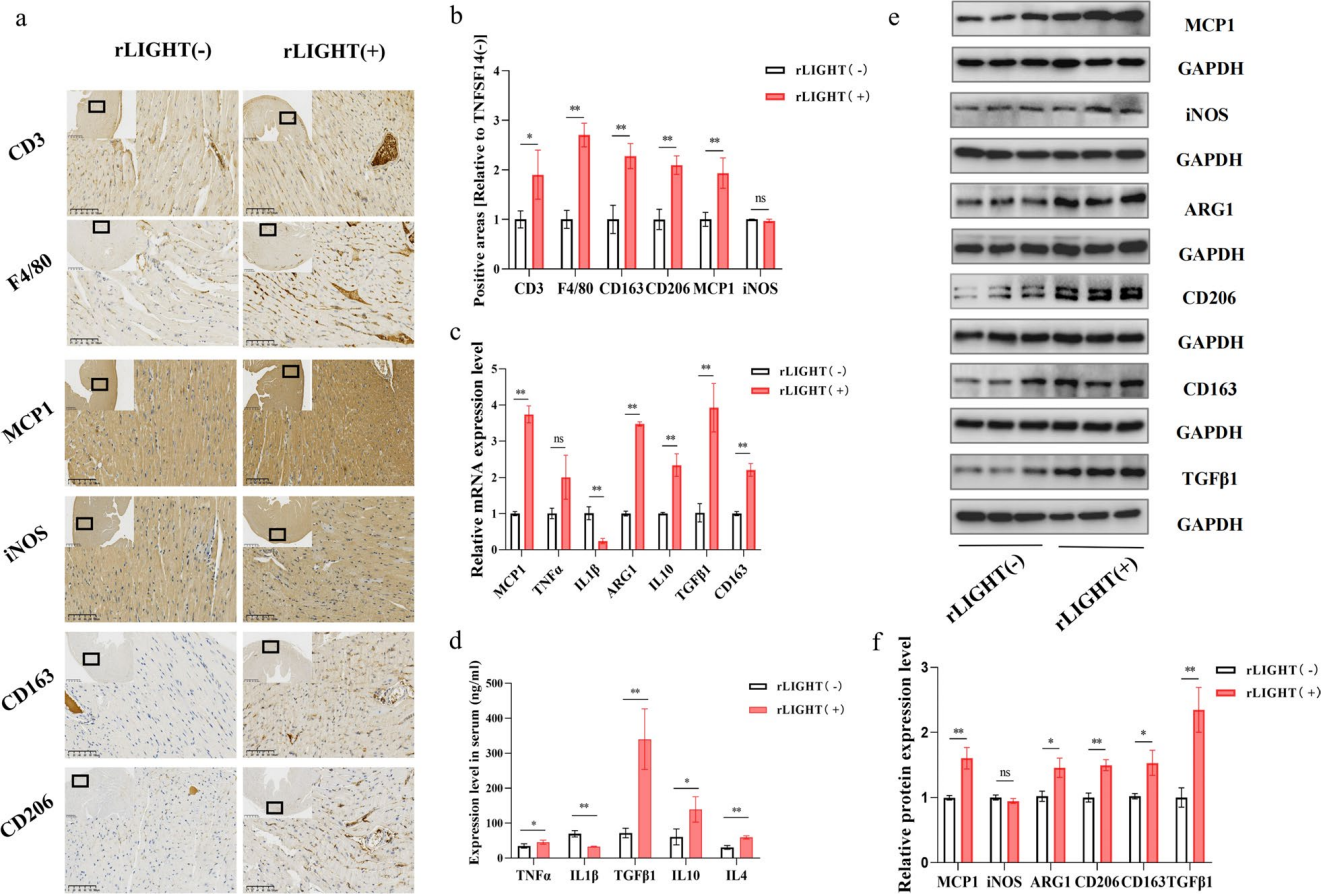
LIGHT contributes to monocyte migration and macrophage M2 polarization. **a** Representative images of mouse cardiac tissues from the different groups with CD3, F4/80, MCP1, iNOS, CD163, and CD206 IHC staining (from above to below). **b** Quantification of CD3^+^, F4/80^+^, MCP1^+^, iNOS^+^, CD163^+^, and CD206^+^ areas in the different groups. **c** mRNA levels of MCP1, TNF-α, IL-1β, IL-10, TGF-β1, ARG1, and CD163 in the different groups [relative to the LIGHT( −) group]. **d** Elevation in the serum levels of TNF-α, IL-1β, TGF-β1, IL-10, and IL4 from different groups. **e** Western blot analysis of MCP1, iNOS, TGF-β1, ARG1, CD163, and CD206, and **f** relative expression in the cardiac tissue normalized to the control. *p < 0.05, **p < 0.01. MCP1: monocyte chemoattractant protein-1; TNF-α: tumour Necrosis Factor α; IL-1β: interleukin 1β; IL-10: interleukin 10; TGF-β1: transforming growth factor β; ARG1: arginase1

The preceding results draw our attention to the potential immunoregulatory ability of LIGHT. Thus, we tested this hypothesis using CD3 and F4/80 IHC staining which, respectively, represent lymphocytes and macrophages, and found that the numbers of both CD3^+^ and F4/80^+^ cells were increased in the LIGHT-treated group (Fig. 4a, b). However, the increasing range of F4/80^+^ cells was larger than that of CD3^+^ cells, suggesting that macrophages may play a more prominent role in LIGHT-induced cardiac remodelling. To investigate the potential function of macrophages in LIGHT-induced cardiac remodelling, we conducted qPCR analysis of macrophage migration- and polarisation-related genes, including MCP1, TNF-α, IL-1β, IL-10, TGF-β1, ARG1, and CD163, and found that the mRNA expression of MCP1, IL-10, TGF-β1, ARG1, and CD163 was increased, while that of IL-1β was decreased, indicating enhanced macrophage migration and M2 polarisation in LIGHT-induced cardiac remodelling (Fig. 4c). Further evidence of macrophage migration in the LIGHT-treated group was confirmed by myocardial IHC (Fig. 4a, b) and western blot analysis for MCP1 (Fig. 4e, f). M2 polarisation was further confirmed by the increased IL4, TGF-β1 and IL-10 levels in LIGHT-treated mouse serum (Fig. 4d) and increased protein expression of ARG1, CD163, and CD206 in LIGHT-treated myocardium, while the M1 polarisation marker iNOS showed no obvious inclination in LIGHT-treated myocardium detected using IHC (Fig. 4a, b) and western blotting (Fig. 4e, f).

### LIGHT contributes to myocardial fibrosis

**Fig. 5:**
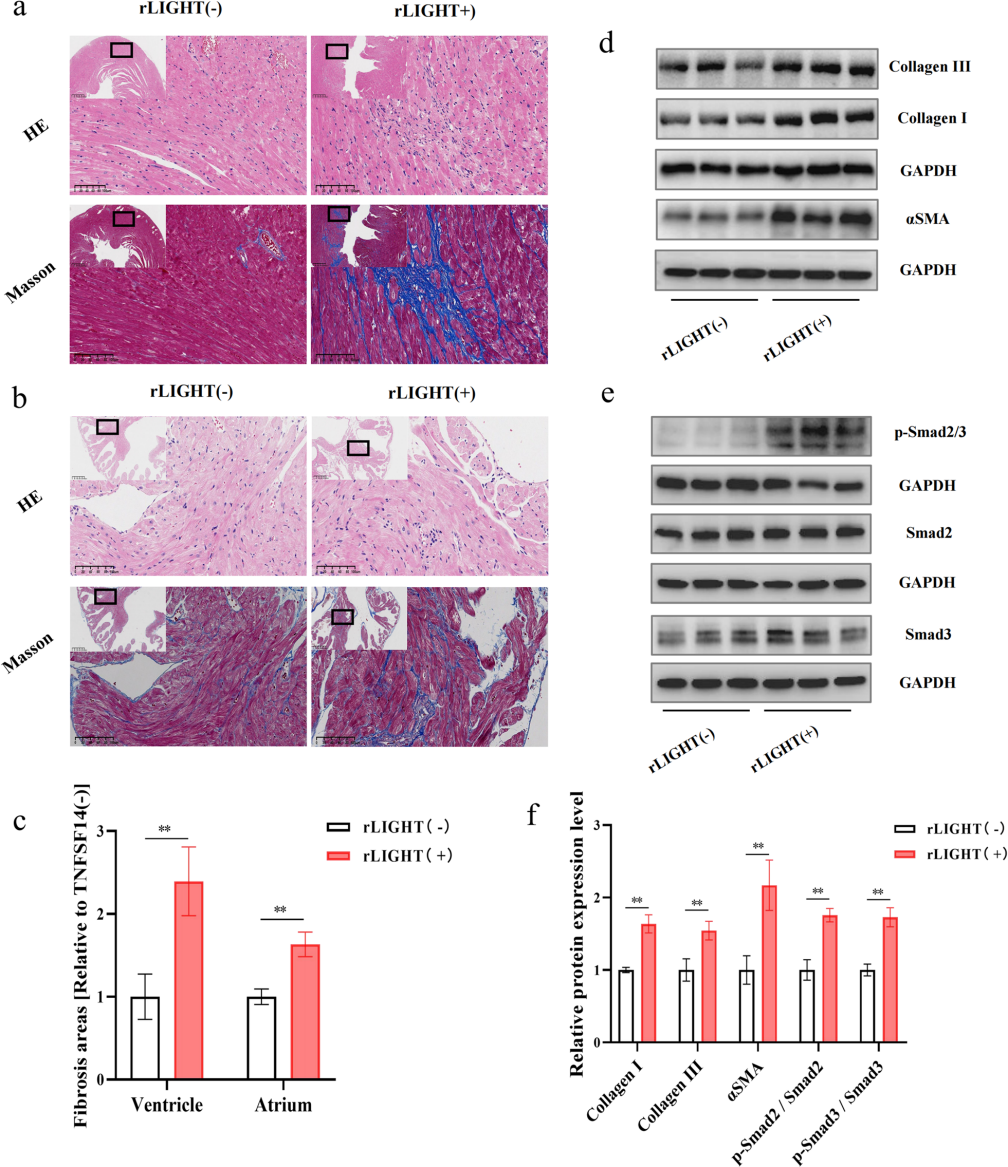
LIGHT contributes to myocardial fibrosis. **a** Typical histological images of the mouse ventricular and **b** atrial tissues from both groups stained with H&E and Masson. **c** Quantification of cardiac fibrosis of ventricular and atrial in the different groups. **d**, **e** Western blot images of collagen I, collagen III, α-SMA, Smad2/3, and phospho-Smad2/3, and **f** relative expression in the heart samples normalized to the control. *p < 0.05, **p < 0.01. α-SMA: α-smooth muscle actin

Undoubtedly, macrophage M2 polarisation could lead to adverse cardiac remodelling, mostly by promoting fibrosis. Thus, cardiac tissue morphology was examined via microscopy using H&E and Masson’s trichrome staining. The ventricle and atria in the LIGHT-treated group showed large disorganized areas indicated by H&E staining (Fig. 5a, b). The total cardiac fibrosis area in the LIGHT-treated group, which was mainly distributed in the myocardial interstitium was enlarged by approximately threefold compared with that in the control group (Fig. 5c). Further verification via western blotting also demonstrated increased collagen deposition and fibroblast-to-myofibroblast transition levels, as revealed by the increased collagen I and III content and α-SMA protein expression (Fig. 5d, f). Moreover, TGF-β1, which is a classical cytokine in regulating fibrosis, was overexpressed in the LIGHT-treated group, indicating that the downstream pathway, including TGF-β/Smad, could be activated under LIGHT stimulation. Further western blot analysis confirmed this hypothesis by revealing increased expression of phosphorylated Smad2/3 (Fig. 5e, f).

### LIGHT regulates M2 polarization via the PI3Kγ/SGK1 pathway

**Fig. 6:**
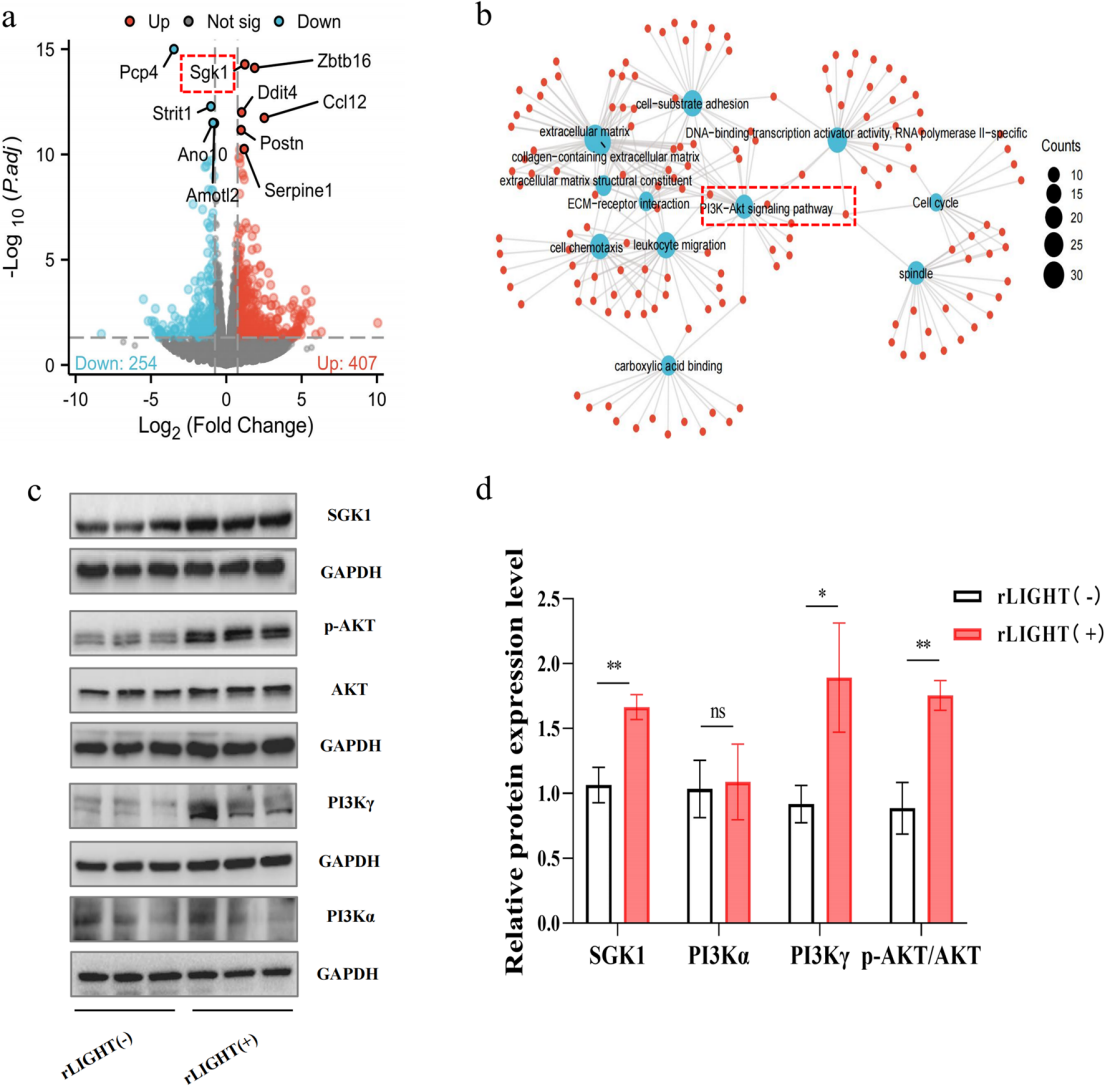
The SGK1 and PI3Kγ pathways were activated in the LIGHT-stimulated heart. **a** Volcano plot of 661 differentially expressed genes. **b** GO and KEGG enrichment analyses of 661 differentially expressed genes. **c** Western blot images of SGK1, AKT, phospho-AKT, and PI3Kα/γ, and **d** relative expression in the heart samples normalized to the control. *p < 0.05, **p < 0.01. SGK1: serum and glucocorticoid-inducible kinase 1; AKT: protein kinase B; PI3Kα/γ: phosphoinositide-3 kinase α/γ

To investigate the mechanism of LIGHT-induced cardiac remodelling, we conducted RNA sequencing of the LIGHT-treated and control groups. A total of 661 DEGs were confirmed (407 upregulated and 254 downregulated) (Fig. 6a). GO enrichment demonstrated that DEGs were enriched in ‘collagen-containing extracellular matrix’, ‘cell chemotaxis’, ‘leukocyte migration’, ‘DNA-binding transcription activator activity’, and ‘RNA polymerase II-specificity’, indicating enhanced immune cell chemotaxis and extracellular matrix deposition (Fig. 6b). Furthermore, KEGG pathway enrichment was observed in the ‘ECM-receptor interaction’, ‘Cell cycle’, and ‘PI3K-Akt signaling pathway’, among which the PI3K-Akt signalling pathway particularly attracted our attention (Fig. 6b). *SGK1* is the key gene in the PI3K-Akt signalling pathway, and it showed significantly increased expression in the LIGHT group at the same time. Thus, we investigated whether there was any difference in SGK1 and PI3Kγ-Akt pathway expression and found that the PI3Kγ-Akt pathway was activated and SGK1 was overexpressed in the LIGHT-induced myocardium, whereas PI3Kα showed no difference (Fig. 6c, d). The most intriguing discovery was that only PI3Kγ, rather than PI3Kα/β/δ, was overexpressed in the LIGHT-treated group (expression data from RNA sequencing), which is crucial for leukocyte recruitment and inflammation [25].

**Fig. 7:**
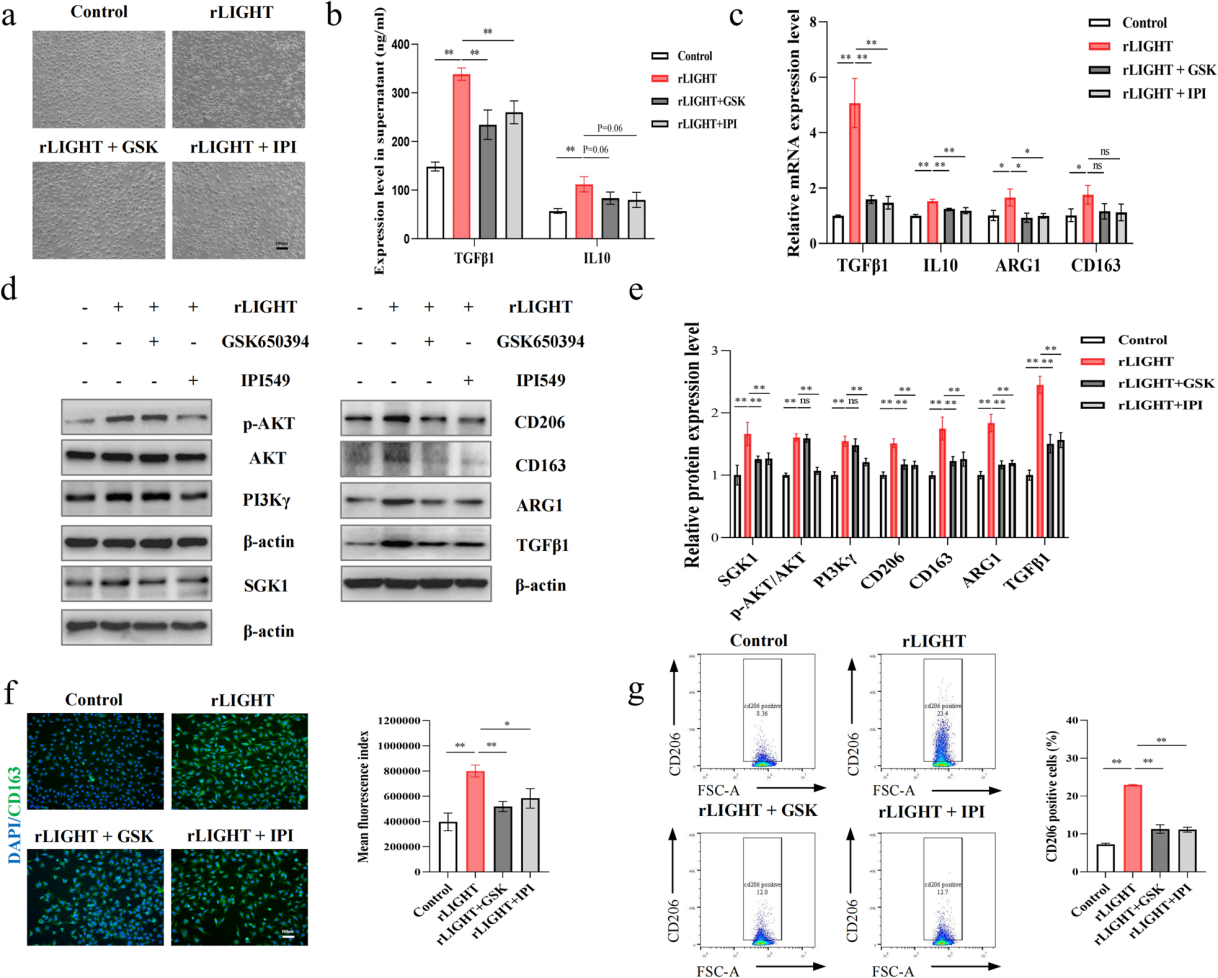
LIGHT promoted BMDM M2 polarization in which PI3Kγ and SGK1 activation are indispensable. **a** Representative morphological images of BMDMs from the different groups. **b** Supernatant protein levels of TNF-α, IL-1β, TGF-β1, and IL-10 in different groups. **c** mRNA levels of IL-10, TGF-β1, ARG1, and CD163 in different groups. **d** Western blot images of SGK1, AKT, phospho-AKT, PI3Kγ, ARG1, CD163, CD206, and TGF-β1, and **e** relative expression in the different groups. **f** Representative fluorescent images of CD163 and the relative fluorescence intensity in different groups. **g** Representative FCM images and the proportion of CD206-positive macrophages in different groups. *p < 0.05, **p < 0.01. IPI: a PI3Kγ inhibitor, IPI549; GSK: a SGK1 inhibitor, GSK650394

Next, we investigated whether the PI3Kγ/SGK1 pathway is involved in LIGHT-induced M2 polarisation. To this end, we used recombinant LIGHT to stimulate BMDM in vitro*.* We found that LIGHT stimulated PI3Kγ and SGK1 overexpression and PI3Kγ mediated AKT phosphorylation along with M2 polarisation marker overexpression both at the mRNA (Additional file 1: Fig. S4a) and protein levels (Additional file 1: Fig. S4b). Furthermore, PI3Kγ inhibitor IPI549 and SGK1 inhibitor GSK650394 were used to investigate whether these inhibitors can rescue LIGHT-induced M2 polarisation in cytotoxicity-free concentrations (Additional file 1: Fig. S5a, b). First, morphological changes also appeared in the LIGHT-treated group, with the BMDMs becoming irregularly round with an extended pseudopod, which could be rescued by PI3Kγ and SGK1 inhibition (Fig. 7a). Next, supernatant protein levels of TGF-β1 and IL-10 were detected and showed significant reduction compared to those in the LIGHT-treated group after PI3Kγ and SGK1 inhibition (Fig. 7b). Moreover, the mRNA (Fig. 7c) and protein (Fig. 7d, e) levels of SGK1, PI3Kγ, phosphorylated AKT, and M2 polarisation markers were reduced in the PI3Kγ and SGK1 inhibitor-treated groups compared to those in the LIGHT-treated group. Notably, the upstream and downstream relationships were illustrated by using the PI3Kγ and SGK1 inhibitors separately. It was found that PI3Kγ inhibition downregulated PI3Kγ and SGK1 simultaneously, while SGK1 inhibition was unable to reduce PI3Kγ protein levels. Immunofluorescence (Fig. 7f) and FCM (Fig. 7g, Additional file 1: Fig. S5c) in BMDMs also showed increased CD163 fluorescence intensity and an increased proportion of CD206-positive macrophages proportion in the LIGHT group, which was rescued by PI3Kγ and SGK1 inhibition.. These results demonstrate that LIGHT induces macrophage M2 polarisation and that PI3Kγ and SGK1 activation is indispensable.

### LIGHT regulates cardiac fibroblast phenotypes via M2 macrophage polarization

**Fig. 8:**
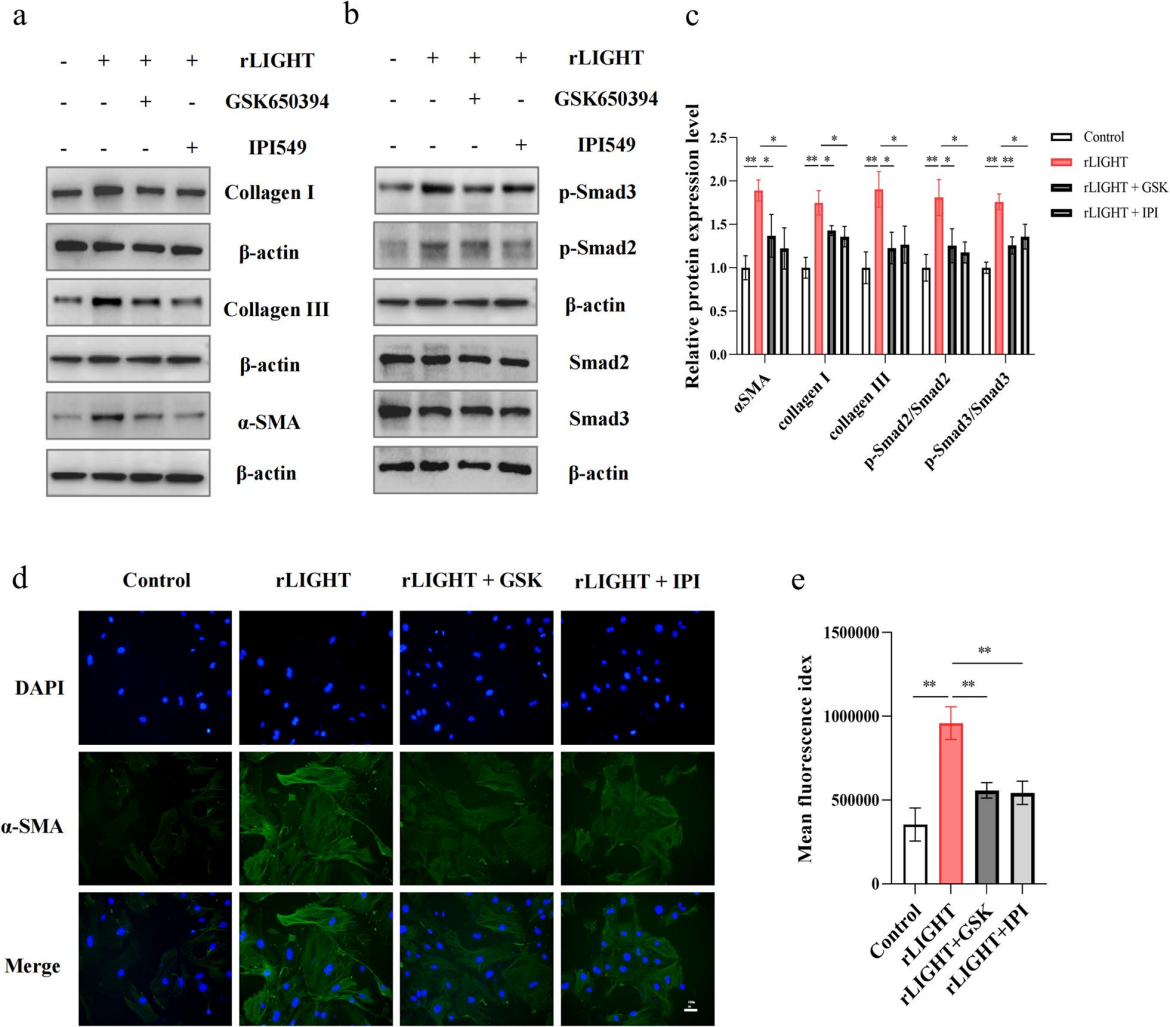
The PI3K and SGK1 inhibitor-treated BMDM CM could rescue CF phenotypes. **a**, **b** Representative western blot images of collagen I, collagen III, Smad2/3, and phospho Smad2/3 and **c** relative expression in different groups. D: Representative fluorescence images of CFs and **g** the relative fluorescence in different groups. *p < 0.05, **p < 0.01

We aimed to determine the detailed mechanism of LIGHT in the regulation of cardiac fibrosis. First, we used recombinant LIGHT to directly stimulate cardiac fibroblasts. However, direct stimulation with recombinant LIGHT did not promote the fibroblast-to-myofibroblast transition and collagen synthesis (Additional file 1: Fig. S6a). Next, we considered whether the cardiac fibroblast phenotypic changes were stimulated by cytokines or secreted proteins from macrophages. Accordingly, we used MCM to culture cardiac fibroblasts. The MCM-treated fibroblasts showed enhanced fibroblast-to-myofibroblast transition and collagen synthesis in the LIGHT-treated group (Additional file 1: Fig. S6b). In addition, treatment with MCM in the PI3Kγ and SGK1 inhibitor-treated groups rescued the changes in CF phenotypes and TGF-β/Smad pathway activation compared to those in the LIGHT group, as assessed via western blotting (Fig. 8a, b, and c) and α-SMA immunofluorescence (Fig. 8d, e). These findings indicate that LIGHT regulates cardiac fibroblast phenotypic changes via M2 macrophage polarisation rather than LIGHT alone.

## Discussion

**Fig. 9:**
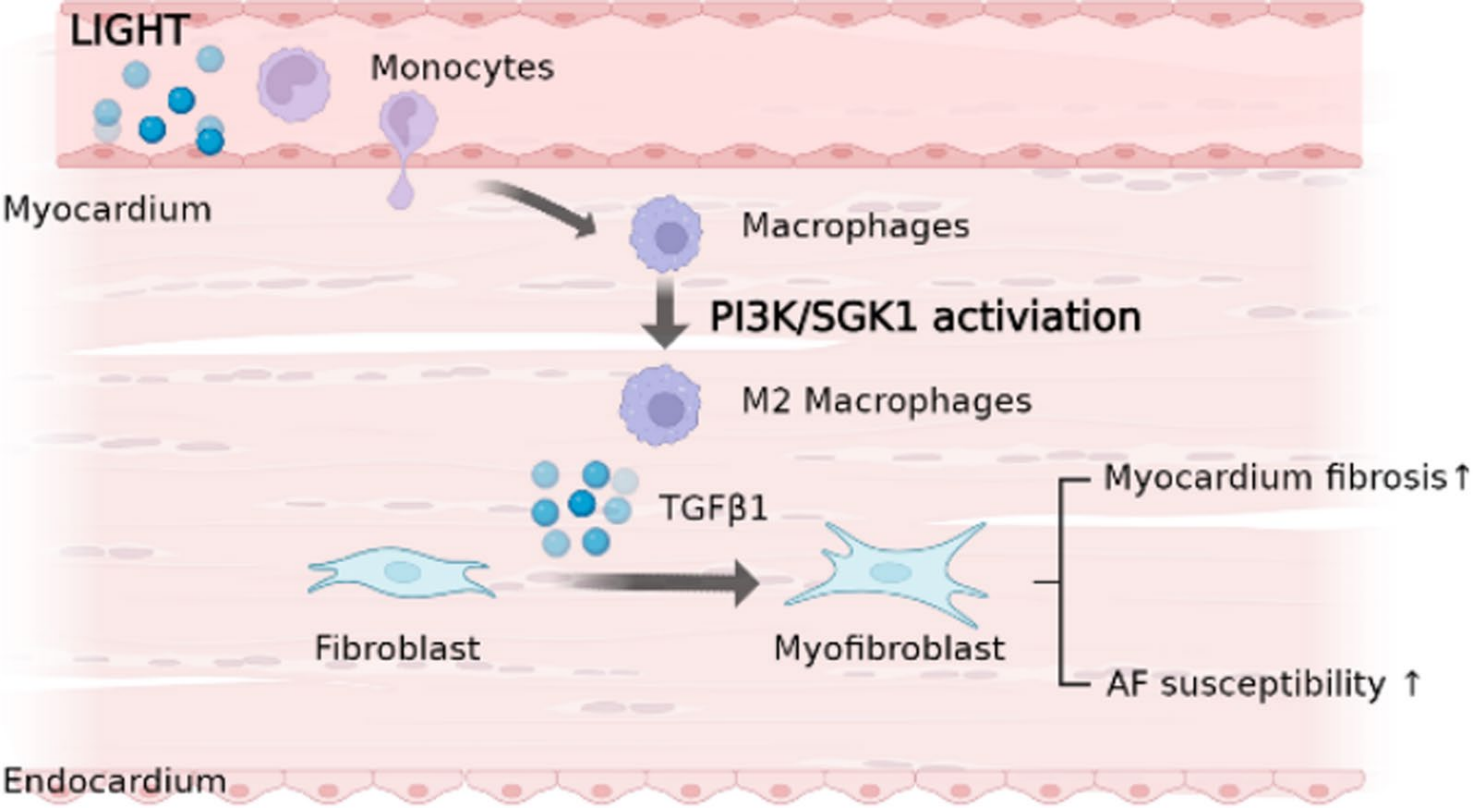
Schematic diagram of the potential effect of LIGHT in promoting cardiac fibrosis and atrial fibrillation vulnerability. LIGHT promoted macrophage migration, infiltration and M2 polarisation both in the ventricle and atrium. TGF-β1 secreted from LIGHT-stimulated macrophages further promoted the fibroblast-to-myofibroblast transition and collagen synthesis which finally cause cardiac fibrosis and atrial fibrillation vulnerability

Here, we conducted studies in patients, animals and cultured cells to address the impact of LIGHT, a novel cytokine ligand the levels of which are significantly elevated in the peripheral blood of patients with AF on the pathogenesis of myocardial fibrosis and AF. Specifically, the expression levels of LIGHT in the peripheral blood of patients with AF were significantly increased, as detected using a PCR array. The correlation between LIGHT and the clinical characteristics of patients with AF was also verified. In vivo and in vitro experiments showed that LIGHT mainly promoted the secretion of cytokines, such as TGF-β1 and IL-10, by inducing macrophage M2 polarisation, thereby increasing the susceptibility to myocardial fibrosis and AF (Fig. 9).

It is commonly recognised that myocardial fibrosis is a cornerstone of the occurrence and maintenance of AF and is involved in both the cardiac fibroblast intrinsic pathway and extrinsic cellular factors [26].In addition, the regulation of the over-activated inflammatory response and crosstalk between fibroblasts and other immune cells have also become novel intervention strategies to target myocardial fibrosis. In our research, we found that LIGHT was mainly expressed by activated lymphocytes (T cells and NK cells) and was significantly elevated in the peripheral blood of AF patients. Lymphocyte activation and the increased secretion of cytokines in AF have been reported as key immune mechanisms in the development and progression of AF and fibrosis [27, 28], and could be the main cause of the elevated LIGHT expression in patients with AF.

In addition to lymphocytes, macrophage recruitment and activation are important factors in the onset of cardiac immune and fibrosis remodelling [29]. Ly6C^hi^ monocytes infiltrate into the heart via MCP1/CCR2 signalling and differentiate into macrophages in the injured myocardium [30]. Several studies have demonstrated a marked reduction in macrophage infiltration and fibrosis in the hearts of MCP1-null mice [31]. Similarly, we noticed significantly increased macrophage infiltration and MCP1 overexpression after intermittent intravenous injection of rLIGHT.

Macrophages are heterogeneous immune cell populations including classically activated (M1) and alternatively activated (M2) macrophages, which are key inflammatory cells in heart disease and are strongly associated with myocardial fibrosis. Evidence suggests that M2 macrophages are activated by glucocorticoids or the T-helper 2 secreted cytokines IL-4, IL-13, and IL-18, leading to the upregulation of IL-10 and TGF-β1, which then facilitates the fibroblast-to-myofibroblast transition characterised by the expression of α-SMA and collagen production [32–36]. In this study, we observed that LIGHT induced M2 macrophage polarisation and cardiac fibrosis in vivo; additionally, in vitro study showed that the expression of TGB-β1 could be stimulated in relatively low rLIGHT conditions, which represents a more obvious fibrotic tendency induced by LIGHT. LIGHT has already been discovered to stimulate macrophage TGF-β1 secretion and promotes the fibroblast-to-myofibroblast transition [12]. In this study, we also observed that rLIGHT-stimulated MCM-treated cardiac fibroblasts showed increased fibroblast-to-myofibroblast transition, collagen synthesis and TGF-β/Smad pathway.

Although few studies have related LIGHT to M2 macrophage polarisation, the ability of LIGHT to induce M2 macrophage polarisation has been implicated in a few studies. Pejman et al. discovered that LIGHT maintains T-helper 2-cell persistence and correspondingly reduces the secretion of several inflammatory cytokines, including IL-4 and IL-13, both of which promote M2 macrophage polarisation [37]. Similar results have been demonstrated in eosinophilic oesophagitis [9] and pulmonary tissue [38]. We also observed elevated expression of IL-4 expression in mouse serum and CD3 + lymphocyte infiltration in myocardial tissue. Additionally, more direct evidence from LIGHT^−/−^ BMDMs has shown diminished IL-10 levels compared to those in the WT controls [11]. This evidence confirmed the ability of LIGHT to promote myocardial fibrosis via M2 polarisation.

In this study, we revealed a new and independent mechanism in LIGHT-induced myocardial fibrosis, wherein LIGHT enhancing the PI3K pathway in cardiac macrophages and upregulated SGK1 levels, thus enhanced M2 macrophage polarisation. The PI3K pathway is a pivotal determinant of cell biology and disease progression, including myocardial fibrosis and atrial fibrillation [39–41]. Moreover, PI3K pathway-mediated macrophage polarisation has been widely investigated. Indeed, M2 polarisation induced by the activated PI3K pathway could lead to the secretion of a large number of fibrotic cytokines, such as TGF-β1 [42, 43]. Notably, recent findings suggest that signalling from PI3Kγ is not only crucial for leukocyte recruitment and inflammation but also contributes to cardiac maladaptive remodelling [25]. Surprisingly, we also noticed the overexpression of PI3Kγ rather than phosphoinositide-3 kinase α/β/δ in the LIGHT group using RNA sequencing.

Furthermore, the intersection between the enriched PI3K pathway genes and significantly DEGs, as determined via RNA sequencing, showed that SGK1 was highly overexpressed in the rLIGHT-stimulated myocardium, which was further verified by western blotting. SGK1 has been widely discovered in the pathophysiological process of cardiac electrophysiological changes. Specifically, the genetic or pharmaceutical inhibition of SGK1 directly inactivates cardiomyocyte sodium channel currents [44], or indirectly rescues obesity or AngII-induced AF by reducing myocardial fibrosis and inflammation [35, 45, 46]. With respects to macrophages SGK1 can not only be recognized as an M2 macrophage marker but also promotes M2 polarisation partly by activating the signal transducer and activator of transcription 3 and forkhead box O1 pathways [35, 47, 48]. Therefore, we focused on whether SGK1 is responsible for the LIGHT-induced M2 polarisation of macrophages and found that LIGHT-induced M2 polarisation could be rescued by utilising PI3Kγ or an SGK1 inhibitor. Similarly, MCM-treated cardiac fibroblasts showed downregulation of the fibroblast-to-myofibroblast transition and collagen aggregation in the PI3Kγ and SGK1 inhibitor groups.

There are some limitations in our study. First, rLIGHT was employed to simulate a high circulating LIGHT level. Our results need to be verified in pathological heart and LIGHT-knockout mouse models. Second, how LIGHT interacts with macrophages via its receptors and which receptors plays a leading role in LIGHT-mediated macrophage migration and polarization remains unknow.

However, our study highlights the cross talk between immune cells (lymphocytes, macrophages) and cardiac stromal cells during myocardial fibrosis and AF. Moreover, LIGHT and its receptors, as co-stimulatory ligands and receptors, are involved in the regulation of lymphocyte function and immune-related systemic and multiorgan dysfunction. In the future, the preparation and clinical translation of monoclonal antibodies against LIGHT or its receptors may be a gospel for patients with cardiovascular diseases.

## Conclusion

In summary, we demonstrated that LIGHT is markedly activated in the peripheral blood of patients with AF with LA remodelling. Importantly, LIGHT primes macrophage polarisation toward the M2 phenotype by activating the PI3Kγ/SGK1 pathway, leading to myocardial fibrosis and AF vulnerability. Altogether, LIGHT could be an attractive target for treating AF and other inflammation- and fibrosis-related cardiovascular diseases.

### Supplementary Information

**Additional file 1.** Supplementary Materials.

## Data Availability

The original data contributing to the findings presented in the study are included in the article/Additional figures. Further inquiries can be addressed to the corresponding author.

## Abbreviations

LIGHT/TNFSF14: Tumour necrosis factor superfamily protein 14
AF: Atrial fibrillation
PBMCs: Peripheral blood mononuclear cells
BMDM: Bone marrow-derived macrophage
MCM: Macrophage-conditioned medium
CFs: Cardiac fibroblasts
HVEM/TNFRSF14: Herpesvirus entry mediator
LTβR: Lymphotoxin β receptor
SR: Sinus rhythm
LVEF: Left ventricle ejection fraction
LVFS: Left ventricle fractional shortening
LA: Left atrium
LVIDd/LVIDs: LV internal dimensions at diastole/systole
NT-proBNP: N-terminal pro brain natriuretic peptide
iNOS: Inducible nitric oxide synthase
DMEM: Dulbecco’s modified Eagle’s medium
M-CSF: Macrophage colony-stimulating hormone
LAD: LA diameter
MCP1: Monocyte chemoattractant protein-1
TNF-α: Tumour necrosis factor α
IL-1β: Interleukin 1β
IL-10: Interleukin 10
TGF-β1: Transforming growth factor β
ARG1: Arginase1
α-SMA: α-Smooth muscle actin
SGK1: Serum and glucocorticoid-inducible kinase 1
AKT: Protein kinase B
PI3K: Phosphoinositide-3 kinase
