# Supplementary material for: TNFSF14/LIGHT promotes cardiac fibrosis and atrial fibrillation vulnerability via PI3Kγ/SGK1 pathway-dependent M2 macrophage polarisation
Source: J Transl Med. 2023 Aug 14;21:544. doi: 10.1186/s12967-023-04381-3 (PMC10424430; doi:10.1186/s12967-023-04381-3)
Supplement: Supplementary file 1 — Additional file 1. Supplementary Materials. [file 12967_2023_4381_MOESM1_ESM.docx]

**Additional information**

**
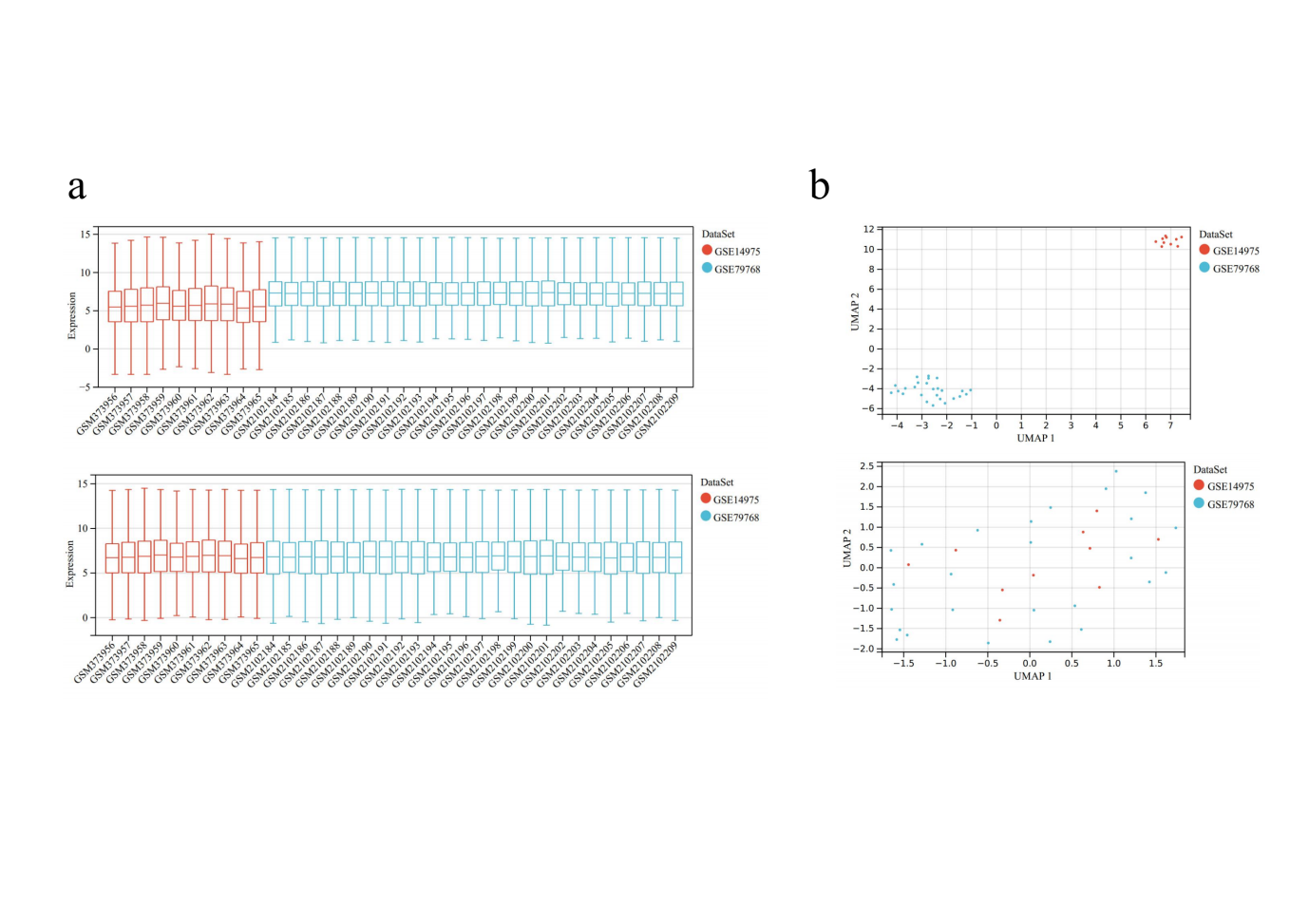
**

Figure S1: Comparison between pre- and post-merged datasets after adjusting and normalizing the batch effect. (a) PCA plot before and after batch effect adjustment and normalization. (b) Boxplot before and after batch effect adjustment and normalization. PCA: principal component analysis.


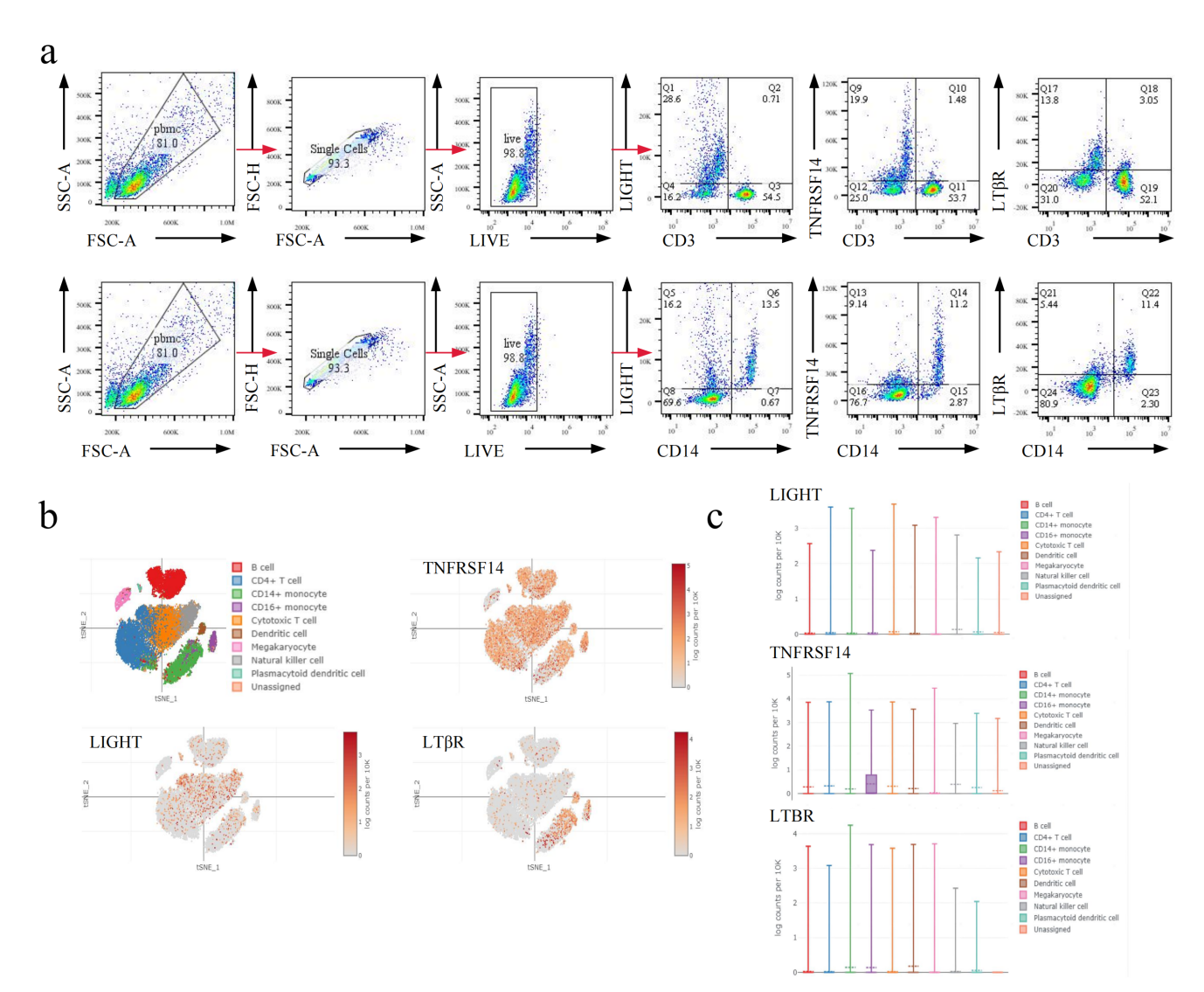


Figure S2: Cell populations distribution of LIGHT and its receptors in PBMCs. (a) Gating strategy and the proportion of TNFRSF14^hi^, LTβR^hi^, and LIGHT^hi^ cells in CD14^+^ or CD3^+^ cells revealed by PBMCs FCM analysis. (b) tSNE map and (c) boxplot of LIGHT, TNFRSF14, and LTβR expression levels in different cell populations revealed by PBMC scRNA sequence data. tSNE: T distributed stochastic neighbour embedding. The relative higher expression cell populations were labeled with red dashed line.


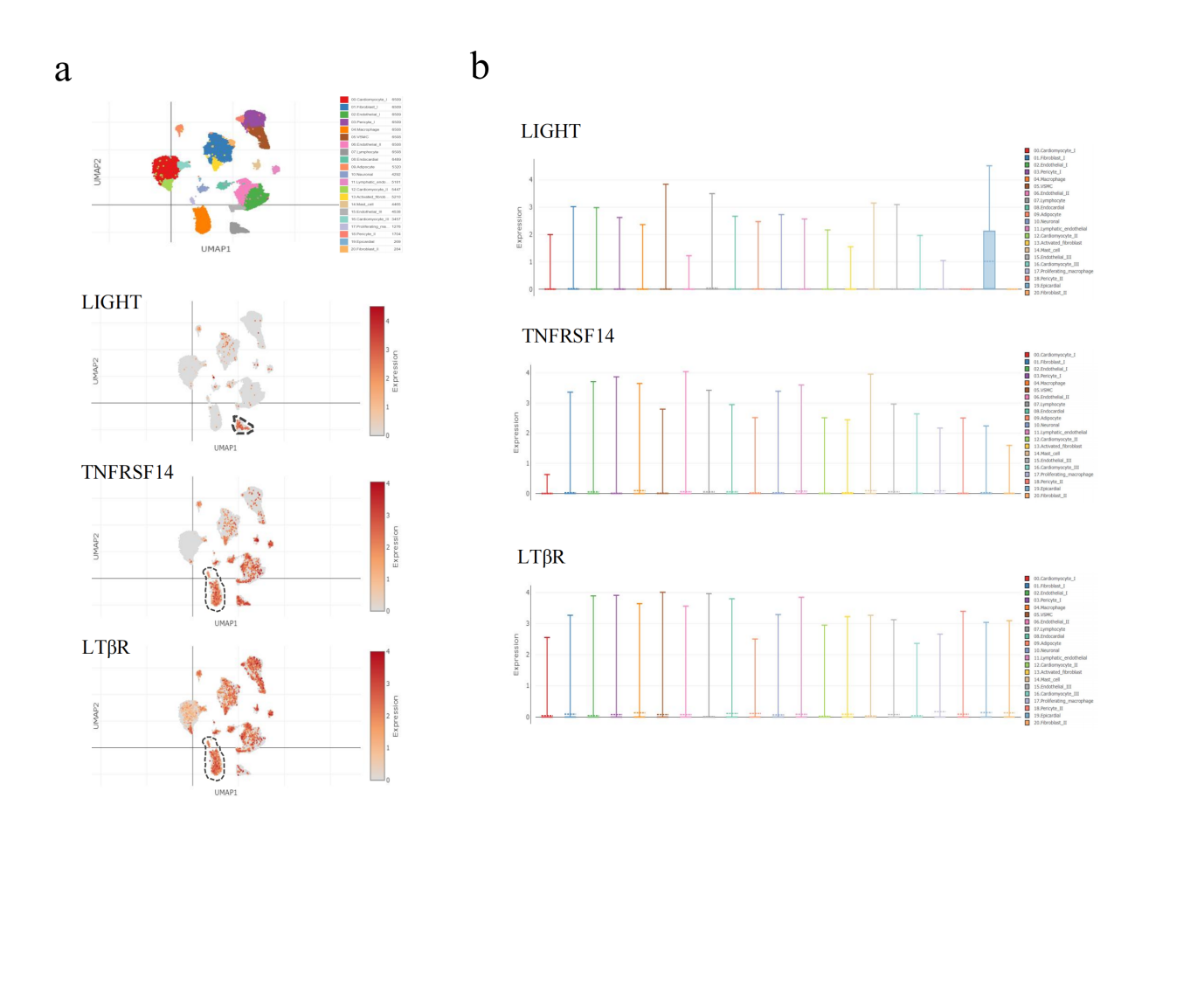


Figure S3:Cell populations distribution of LIGHT and its receptors in myocardium. (a) UMAP and (b) boxplot of LIGHT, TNFRSF14, and LTβR expression levels in different cell populations revealed by myocardium scRNA sequence data.


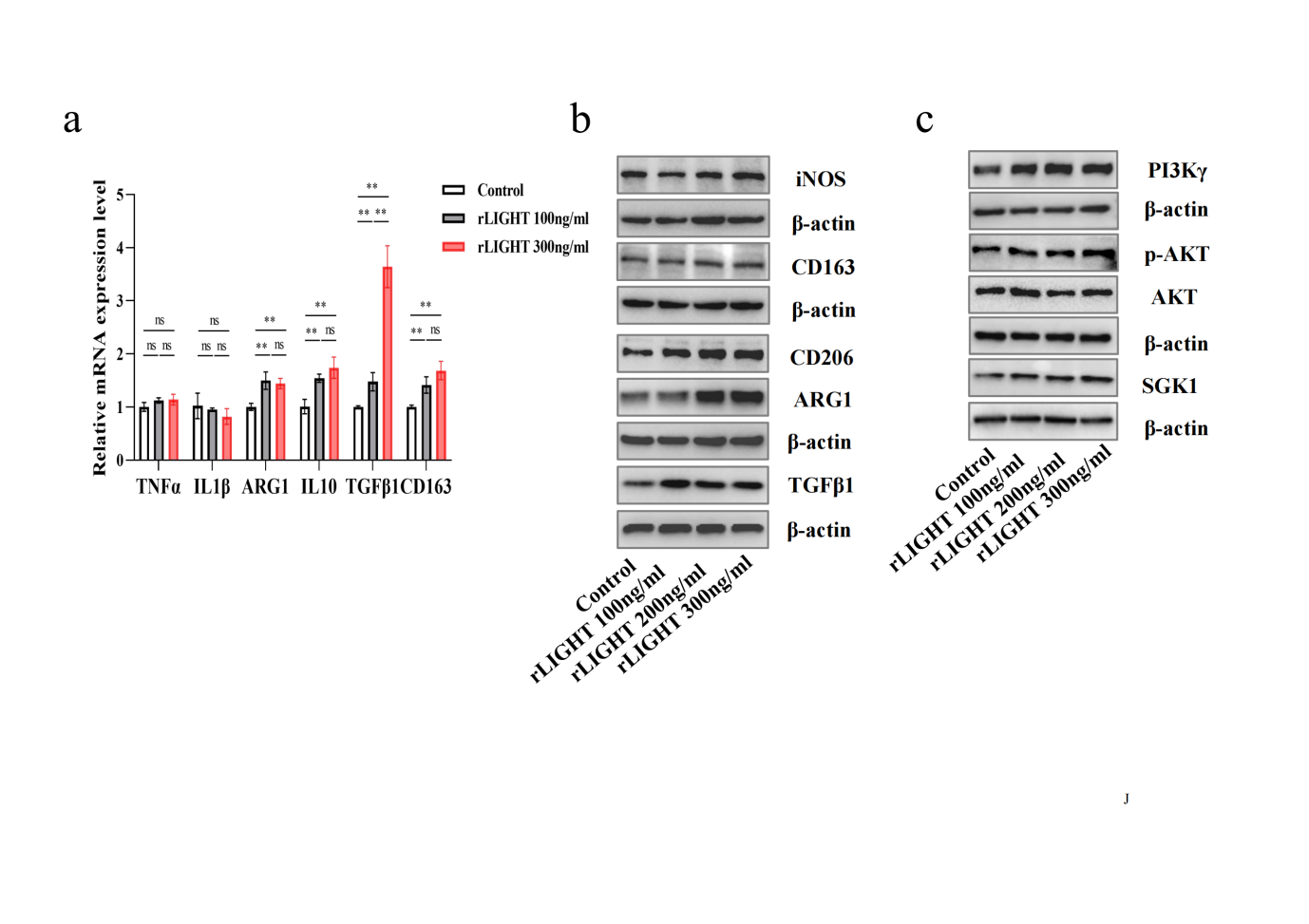


Figure S4: LIGHT promotes M2 macrophages polarisation and PI3Kγ/SGK1 pathway activation in vitro. (a) mRNA levels of MCP1, TNF-α, IL-1β, IL-10, TGF-β1, ARG1, and CD163 in different groups. (b) Western blot analysis of ARG1, CD163, CD206, TGF-β1, and iNOS and (c) SGK1, AKT, phospho-AKT, PI3Kγ in different groups.


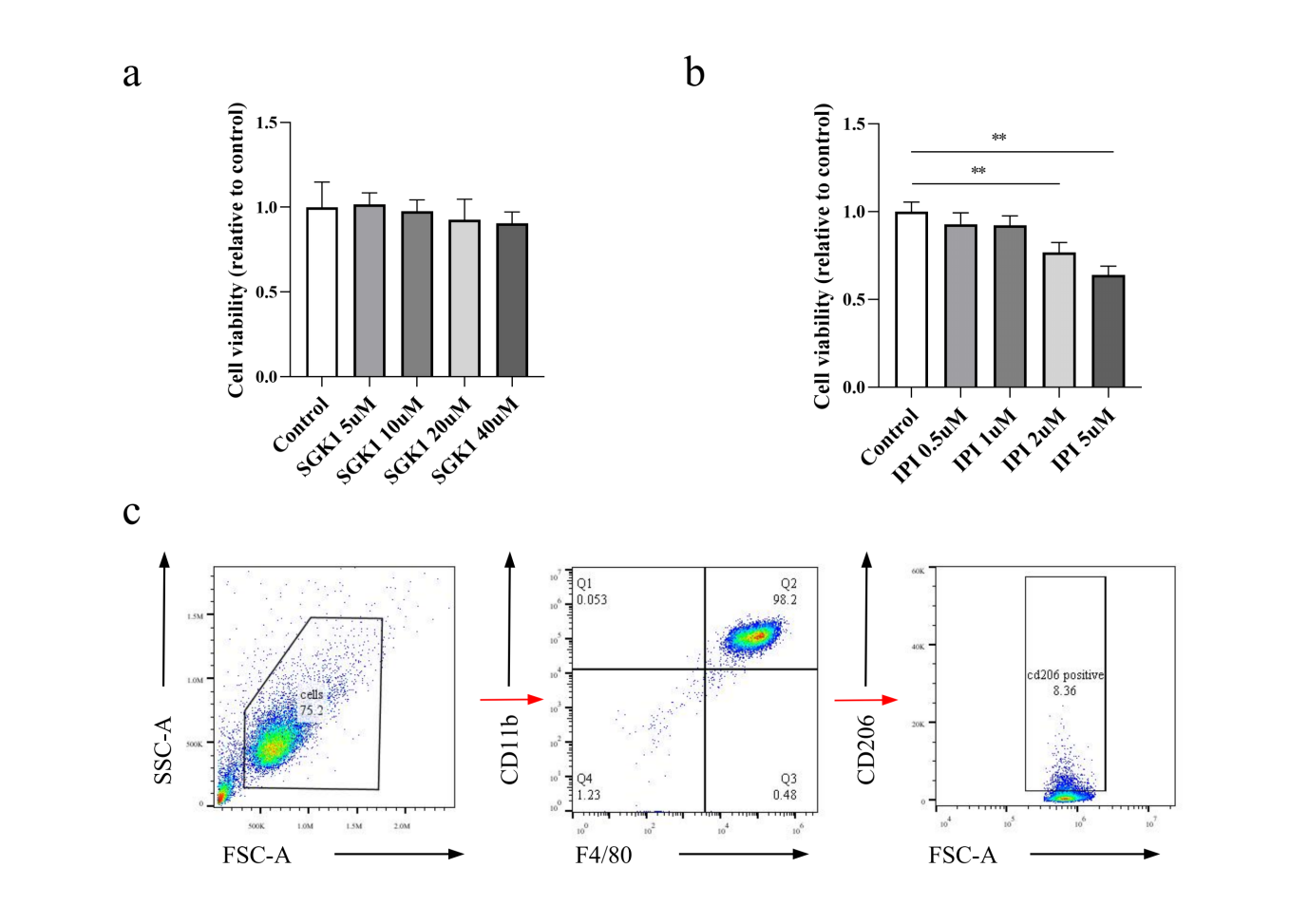


Figure S5: (a) The cytotoxicity of PI3Kγ inhibitor and (b) SGK1 inhibitor detected by CCK8. (c) Gating strategy of CD206-positive macrophages. *p < 0.05, **p < 0.01.


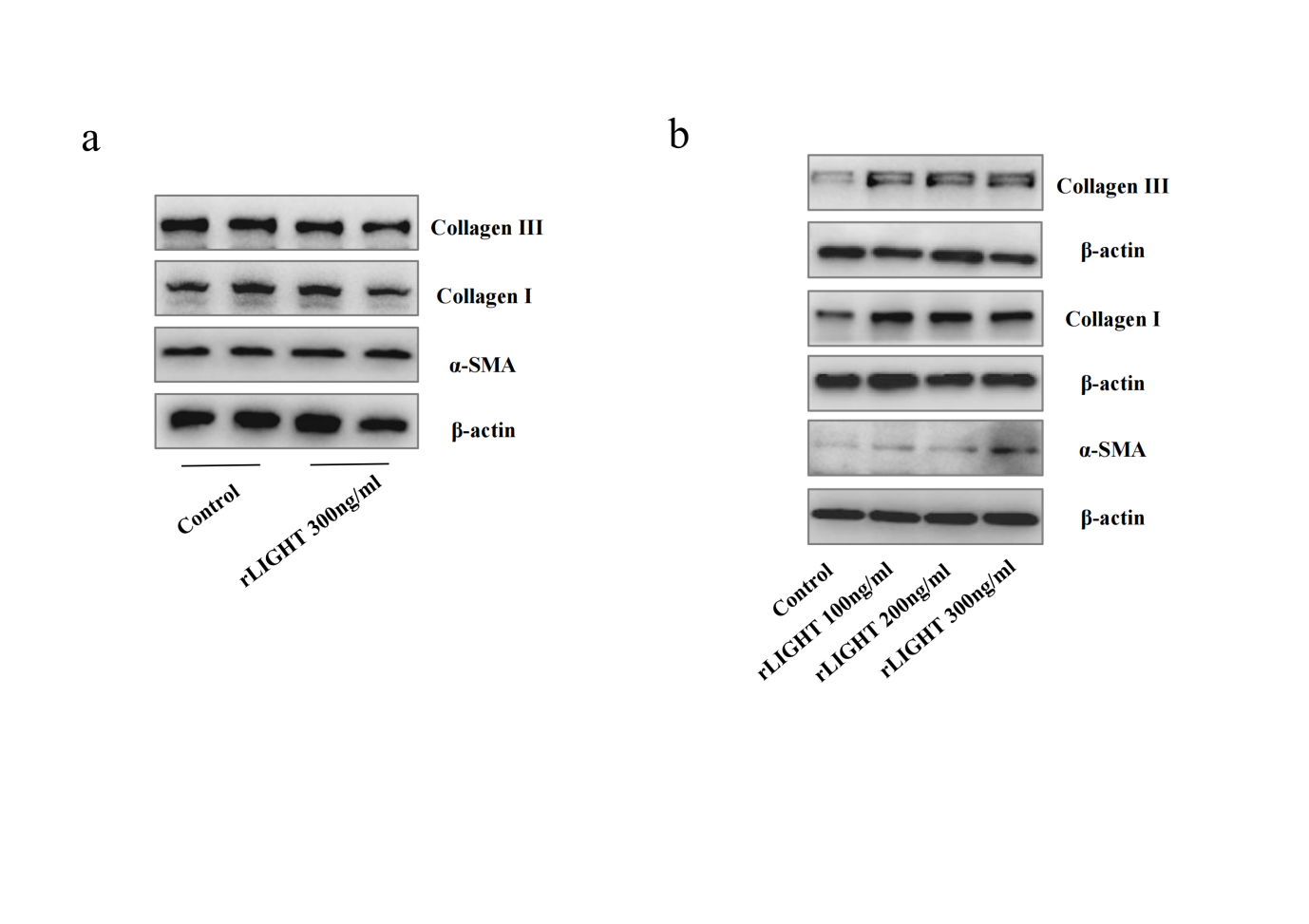


Figure S6: LIGHT regulates CF phenotypes via M2 macrophages polarisation. (a) Representative western blot images of collagen I, collagen III, and αSMA after direct LIGHT stimulation in CFs. (b) Representative western blot images of collagen I and collagen III and αSMA after LIGHT-stimulated BMDMs CM culture of CFs.

Table S1 Primer sequences used for q RT-PCR.

| Gene (Mus) | Forward primer (5’-3’) | Reverse primer (5’-3’) |
| --- | --- | --- |
| ARG1 | AGACAGCAGAGGAGGTGAAGAGTAC | AAGGTAGTCAGTCCCTGGCTTATGG |
| MCP1 | CACTCACCTGCTGCTACTCATTCAC | CTTCTTTGGGACACCTGCTGCTG |
| CD163 | AGAATCACATCATGGCACAGGTCAC | ACAGGAGGAAGACAATGAGGAGGAC |
| TNFα | CGCTCTTCTGTCTACTGAACTTCGG | GTGGTTTGTGAGTGTGAGGGTCTG |
| TGFβ1 | ACCGCAACAACGCCATCTATGAG | GGCACTGCTTCCCGAATGTCTG |
| IL1β | TGCCACCTTTTGACAGTGATG | TGATACTGCCTGCCTGAAGC |
| IL10 | TGCCAAGCCTTATCGGAAATGATCC | AGCCGCATCCTGAGGGTCTTC |
| GAPDH | GCCAAAAGGGTCATCATCTC | GGCCATCCACAGTCTTCT |

Table S2 The Characteristics of patients involved in PCR array.

|  | SR(n=3) | AF(n=4) | P value |
| --- | --- | --- | --- |
| Characteristics |  |  |  |
| Basic profiles |  |  |  |
| Age, y | 64±10.81 | 66.25±9.74 | 0.827 |
| Men,n(%) | 2(66.6) | 2(50) | 0.629 |
| BMI,kg/m2 | 23.93±4.39 | 27.05±1.82 | 0.279 |
| Smokers,n(%) | 0(0) | 1(25) | 0.571 |
| Alcohol,n(%) | 0(0) | 1(25) | 0.571 |
|  |  |  |  |
| Comorbidities |  |  |  |
| Hypertension,n(%) | 2(66.6) | 2(50) | 0.629 |
| Diabetes mellitus,n(%) | 0(0) | 1(25) | 0.571 |
| Ischemic stroke,n(%) | 0(0) | 0(0) | 1.000 |
|  |  |  |  |
| Baseline lab data |  |  |  |
| TG,mmol/l | 1.65±0.67 | 1.92±0.49 | 0.735 |
| LDL-C,mmol/l | 2.66±1.24 | 2.90±0.76 | 0.526 |
| GFR,ml/min | 78.33±1.53 | 82.75±8.92 | 0.081 |
| Albumin, g/L | 42.73±1.51 | 40.88±4.98 | 0.113 |
| Hemoglobin, g/L | 146.67±20.79 | 141.75±21.64 | 0.951 |
| Platelet count (10^9/L) | 159±64.51 | 189.25±59.87 | 0.852 |
| WBC (10^9/L) | 5.87±0.91 | 6.08±1.64 | 0.405 |
| CRP, mg/L | 2.2±1.22 | 1.33±1.20 | 0.917 |
|  |  |  |  |
| Cardiovascular profiles |  |  |  |
| AF types |  |  | 0.029 |
| Paroxysmal,n(%) | 0(0) | 0(0) |  |
| Persistent,n(%) | 0(0) | 4(100) |  |
| LVEF, % | 71.33±6.66 | 61.68±3.97 | 0.351 |
| LVDId,cm | 5.09±0.17 | 4.76±0.35 | 0.309 |
| LAD,cm | 4±0.44 | 4.48±0.19 | 0.096 |
| NT-proBNP,pg/ml | 272.73±209.68 | 536.25±381.89 | 0.386 |
|  |  |  |  |
| Drugs |  |  |  |
| β-blocker | 1(33.3) | 2(50) | 0.629 |
| ACE1/ARB |  |  | 0.143 |
| ACEI,n(%) | 0(0) | 1(25) |  |
| ARB,n(%) | 2(66.6) | 0(0) |  |
| Oral anticoagulants |  |  | 0.029 |
| Warfarin,n(%) | 0(0) | 2(50) |  |
| NOAC,n(%) | 0(0) | 2(50) |  |
| Antiarrhythmic drugs | 0(0) | 1(25) | 0.571 |

Data were means ± SD for skewed variables or proportions for categorical variables. BMI: body mass index; TG: triglyceride; LDL-C: low-density lipoprotein cholesterol; GFR: glomerular filtration rate; WBC: white blood cells; CRP: c-reactive protein; LAD: left atrial diameter; NT-proBNP: N-terminal pro brain natriuretic peptide; ACEI: angiotensin-converting enzyme inhibitors; ARB: angiotensin receptor blockers; NOAC: novel oral anticoagulants.

Table S3 The Characteristics of patients involved in LIGHT ELISA tests.

|  | SR(n=16) | AF(n=26) | P value |
| --- | --- | --- | --- |
| Characteristics |  |  |  |
| Basic profiles |  |  |  |
| Age,y | 55.06±13.82 | 64.19±13.05 | 0.037 |
| Men,n(%) | 8(50) | 19(73.1) | 0.119 |
| BMI,kg/m2 | 24.26±2.92 | 24.08±2.63 | 0.830 |
| Smokers,n(%) | 2(12.5) | 8(30.8) | 0.165 |
| Alcohol,n(%) | 4(25) | 9(34.6) | 0.382 |
|  |  |  |  |
| Comorbidities |  |  |  |
| Hypertension,n(%) | 8(50) | 16(61.5) | 0.339 |
| Diabetes mellitus,n(%) | 2(12.5) | 5(19.2) | 0.454 |
| Ischemic stroke,n(%) | 0(0) | 1(3.85) | 0.619 |
|  |  |  |  |
| Baseline lab data |  |  |  |
| TG,mmol/l | 1.43±0.53 | 1.30±0.48 | 0.390 |
| LDL-C,mmol/l | 2.42±0.73 | 2.20±0.78 | 0.359 |
| GFR,ml/min | 88.75±10.66 | 82.92±13.68 | 0.154 |
| Albumin, g/L | 39.28±3.43 | 40.99±7.74 | 0.163 |
| Hemoglobin, g/L | 137.38±12.49 | 136.35±13.04 | 0.986 |
| Platelet count (10^9/L) | 209.19±49.90 | 198.42±62.75 | 0.437 |
| WBC (10^9/L) | 5.89±0.96 | 6.40±1.92 | 0.039 |
| CRP, mg/L | 2.41±0.98 | 6.62±12.94 | 0.039 |
|  |  |  |  |
| Cardiovascular profiles |  |  |  |
| AF types |  |  |  |
| Paroxysmal,n(%) | 0(0) | 16(61.5) |  |
| Persistent,n(%) | 0(0) | 10(38.5) |  |
| LVEF, % | 68±4.4 | 62±6.1 | 0.001 |
| LVDId,cm | 4.71±0.30 | 4.96±0.49 | 0.064 |
| LAD,cm | 3.62±0.45 | 4.47±0.68 | 0.000 |
| NYHA classification |  |  | 0.044 |
| Class I,n(%) | 16(100) | 20(76.92) |  |
| Class II,n(%) | 0(0) | 6(23.08) |  |
| NT-proBNP,pg/ml | 177.7±167.64 | 814.85±923.46 | 0.009 |
|  |  |  |  |
| Drugs and treatment |  |  |  |
| β-blocker | 4(50) | 15(57.7) | 0.039 |
| ACE1/ARB |  |  | 0.290 |
| ACEI,n(%) | 1(6.3) | 4(15.4) |  |
| ARB,n(%) | 0(0) | 10(38.5) |  |
| Oral anticoagulants |  |  | 0.000 |
| Warfarin,n(%) | 0(0) | 19(73.1) |  |
| NOAC,n(%) | 0(0) | 7(26.9) |  |
| Oral antiplatelet | 2(12.5) | 0(0) | 0.139 |
| Antiarrhythmic drugs | 0(0) | 15(57.69) | 0.000 |

Data were means±SD for skewed variables or proportions for categorical variables.

Table S4 Univariate logistic regression analysis in diagnosing AF

| variables | *P* | *OR* | *95%CI* |
| --- | --- | --- | --- |
| gender(0:female,1:male) | 0.135 | 2.714 | 0.734-10.041 |
| age (year） | 0.048 | 1.052 | 1-1.107 |
| BMI (kg/m2） | 0.827 | 0.974 | 0.772-1.229 |
| smoke(0:health,1:smoke) | 0.191 | 3.111 | 0.569-17.024 |
| Alcohol(0:health,1:Alcohol) | 0.514 | 1.588 | 0.395-6.379 |
| Hypertension(0:health,1:disease) | 0.464 | 1.600 | 0.454-5.634 |
| Diabetes(0:health,1:disease) | 0.572 | 1.667 | 0.283-9.822 |
| Stroke(0:health,1:disease) | 1.000 | 1033903899.425 | 0- |
| NYHA classification（CLASS I:0,CLASS II:1） | 0.999 | 1292379874.281 | 0- |
| TG (mmol/l） | 0.382 | 0.565 | 0.157-2.034 |
| LDL-C (mmol/l） | 0.352 | 0.673 | 0.292-1.550 |
| NT-proBNP (pg/ml） | 0.008 | 1.006 | 1.002-1.010 |
| GFR( ml/min） | 0.161 | 0.963 | 0.913-1.015 |
| LVEF (%） | 0.006 | 0.000 | 0-0.003 |
| LVIDd (cm） | 0.071 | 4.215 | 0.884-20.100 |
| LAD(mm） | 0.002 | 1.336 | 1.109-1.610 |
| LIGHT( ng/ml） | 0.018 | 1.129 | 1.021-1.248 |
| CRP (mg/L） | 0.114 | 1.775 | 0.872-3.612 |
| WBC (10^9/L） | 0.328 | 1.241 | 0.805-1.913 |
| Hemoglobin (g/L） | 0.797 | 0.994 | 0.946-1.044 |
| Platelet (10^9/L） | 0.555 | 0.997 | 0.986-1.008 |
| Albumin (g/L） | 0.411 | 1.050 | 0.935-1.178 |
| β-blocker(0:non,1:β-blocker) | 0.044 | 4.091 | 1.036-16.152 |
| ACEI/ARB（non:0,ACEI:1,ARB:2） | 0.636 | 1.375 | 0.367-5.146 |

OR: odds ratios; 95%CI: 95%confidence interval.

Table S5 Multivariate logistic regression analysis in diagnosing AF

| variables | P | OR | 95%CI |
| --- | --- | --- | --- |
| age(year) | 0.077 | 0.898 | 0.797-1.012 |
| NT-proBNP(pg/ml) | 0.040 | 1.011 | 1.001-1.021 |
| LVEF(%) | 0.080 | 0.000 | 0.000-50.108 |
| LAD(mm) | 0.021 | 1.615 | 1.076-2.425 |
| LIGHT(ng/ml) | 0.434 | 1.085 | 0.884-1.333 |
| β-blocker(0:non,1:β-blocker) | 0.581 | 2.721 | 0.078-95.156 |

OR: odds ratios; 95%CI: 95%confidence interval.
